# The effect of adverse childhood experiences on chronic pain and major depression in adulthood: a systematic review and meta-analysis

**DOI:** 10.1016/j.bja.2023.03.008

**Published:** 2023-04-21

**Authors:** Georgia Antoniou, Emilie Lambourg, J. Douglas Steele, Lesley A. Colvin

**Affiliations:** 1 Division of Population Health and Genomics, Medical Research Institute, University of Dundee, Dundee, UK; 2 Division of Imaging Science and Technology, Medical School, University of Dundee, Dundee, UK

**Keywords:** adverse childhood experiences, chronic pain, depression, early life adversity, major depressive disorder, MRI, neuroimaging, neuropathic pain

## Abstract

**Background:**

Adverse childhood experiences have been linked to increased multimorbidity, with physical and mental health consequences throughout life. Chronic pain is often associated with mood disorders, such as major depressive disorder (MDD); both have been linked to adverse childhood experiences. It is unclear how the effect of adverse childhood experiences on neural processing impacts on vulnerability to chronic pain, MDD, or both, and whether there are shared mechanisms. We aimed to assess evidence for central neural changes associated with adverse childhood experiences in subjects with chronic pain, MDD, or both using systematic review and meta-analysis.

**Methods:**

Electronic databases were systematically searched for neuroimaging studies of adverse childhood experiences, with chronic pain, MDD, or both. Two independent reviewers screened title, abstracts, and full text, and assessed quality. After extraction of neuroimaging data, activation likelihood estimate meta-analysis was performed to identify significant brain regions associated with these comorbidities.

**Results:**

Forty-nine of 2414 studies were eligible, of which 43 investigated adverse childhood experiences and MDD and six investigated adverse childhood experiences and chronic pain. None investigated adverse childhood experiences, chronic pain, and MDD together. Functional and structural brain abnormalities were identified in the superior frontal, lingual gyrus, hippocampus, insula, putamen, superior temporal, inferior temporal gyrus, and anterior cerebellum in patients with MDD exposed to adverse childhood experiences. In addition, brain function abnormalities were identified for patients with MDD or chronic pain and exposure to adverse childhood experiences in the cingulate gyrus, inferior parietal lobule, and precuneus in task-based functional MRI studies.

**Conclusions:**

We found that adverse childhood experiences exposure can result in different functional and structural brain alterations in adults with MDD or chronic pain compared with those without adverse childhood experiences.

**Systematic review protocol:**

PROSPERO CRD42021233989.

**Editor's key points**

• The association between adverse childhood experiences and chronic pain or depression has been well established, but the neural substrates are poorly understood.
• This systematic review with meta-analysis of the long-term effects of adverse childhood experiences on chronic pain and depression identifies limited literature examining the neural correlates of chronic pain and adverse childhood experiences, with no studies of adverse childhood experiences and comorbid chronic pain and depression.
• The authors highlight the need for further research to improve understanding of the neural mechanisms underlying chronic pain, adverse childhood experiences, and depression.

Chronic pain is defined as pain that lasts longer than 3 months, or pain that persists beyond normal healing time.^1^ The prevalence of chronic pain in the UK is 35–53% of the population, increasing with older age, and often associated with comorbid conditions such as depression.^2^, ^3^, ^4^, ^5^ A recent systematic review found that over half of patients with fibromyalgia had co-existing major depressive disorder (MDD),^6^, ^7^, ^8^ although the nature of the link between pain and depression is not clear.^7^ At a global level, chronic pain conditions are the leading cause of years lived with disability,^9^ and, independent of pain, MDD has a prevalence of approximately 10%.^10^, ^11^, ^12^

Adverse childhood experiences (adverse childhood experiences) are defined as repeated aversive experiences that represent a deviation from the expected environment and require adaptation.^13^^,^^14^ Examples include physical, sexual, and emotional abuse, parental illness, criminality, violence, neglect, and poverty.^13^, ^14^, ^15^, ^16^, ^17^ Chronic pain has been found to be related to adverse childhood experiences^18^; for example exposure to adverse childhood experiences may be a risk factor for fibromyalgia in later life.^19^ As with chronic pain, adverse childhood experiences have been proposed as a risk factor for developing mood disorders later in life.^20^^,^^21^ Adverse childhood experiences are associated with elevated risk for multimorbidity with both mental and physical health problems. Although reasons for this are poorly understood, long-term immune system and neurobiological changes may play a role in altering vulnerability to chronic pain and MDD in adult life.^22^^,^^23^

The quality of parental care, nutrition, cognitive stimulation, and socioeconomic status during early child development have been shown to affect brain morphology and functionality throughout the life course.^24^ Neuroimaging is an important tool in advancing understanding of the neural correlates of chronic pain^25^ and depression, and allows exploration of the impact of adverse childhood experiences, with the potential to identify common mechanisms.^24^^,^^26^, ^27^, ^28^ There have been some neuroimaging studies of chronic pain and adverse childhood experiences identifying involvement of various brain regions, such as the insular cortex, basal ganglia, amygdala, hippocampal cortex, and prefrontal cortex^16^^,^^17^^,^^26^; however these studies had not examined the comorbidity of adverse childhood experiences and chronic pain. Adverse childhood experiences and MDD neuroimaging studies share several common features, with reported abnormalities of the hippocampus, amygdala, anterior insular, and superior temporal gyrus,^29^, ^30^, ^31^ but these studies had revealed the abnormalities without the comorbidity of adverse childhood experiences and MDD. A notable brain region strongly implicated as affected by adverse childhood experiences and depression in neuroimaging studies is the hippocampus; specifically, there is robust evidence for a smaller hippocampal volume.^30^^,^^32^, ^33^, ^34^, ^35^, ^36^, ^37^, ^38^ There is limited understanding of how comorbid chronic pain and MDD are affected by adverse childhood experiences.

In this systematic review and meta-analysis, we aimed to synthesise evidence from relevant neuroimaging studies to investigate the impact of adverse childhood experiences on the neural correlates of chronic pain, and depression, both alone and in combination.

## Methods

### Literature

After prospective registration on PROSPERO (https://www.crd.york.ac.uk/prospero/display_record.php?RecordID=233989; Registration number: CRD42021233989), a systematic literature search, data extraction, and meta-analysis were carried out. The search was conducted using the following electronic databases, MEDLINE (OVID), Embase (OVID), the Cochrane Central Registry of Controlled Trials (CENTRAL) (the Cochrane Library), PsycINFO (OVID), PubMed, Neurosynth, Sleuth, and Web of Science. An advanced search strategy was developed using a list of MeSH and keywords in combination or alone, such as chronic pain, neuropathic pain, MDD, depression, anxiety, neuroimaging, MRI, and early life adversity. Searches were customised for each database. The searches were restricted to English language, whereas no publication status, or date restrictions were imposed on the systematic search. Searches were imported into Rayyan for removal of duplicates, screening, and study selection (https://www.rayyan.ai/).

### Eligibility criteria

Eligible studies were on humans, were randomised or non-randomised, or observational (cross-sectional or longitudinal, cohort). To be eligible for review, studies had to include participants with chronic pain, depression with a history of adverse childhood experiences, or both, and have quantitative data and neuroimaging measures (i.e. structural and functional MRI), positron emission tomography (PET) or single-photon emission computed tomography (SPECT). Participants/study population of selected papers were adults aged 16 yr or older. Chronic pain was defined as pain that had been present for at least 3 months, and adverse childhood experiences were defined as repeated aversive experiences that represent a deviation from the expected environment and require adaptation and had been identified by a questionnaire such as the Childhood Trauma Questionnaire (CTQ).^39^ Standard depression rating scales^40^, ^41^, ^42^ were converted to a summary measure using a recognised conversion table in order to allow comparisons.^42^ Studies were excluded if patients had acute pain, there was no diagnosis of depression, or depression severity was mild. Studies involving participants with brain injury, brain tumours, stroke, or neurodegenerative conditions were also excluded.

### Study selection

To investigate the associations between chronic pain, mood disorder, and the effects of adverse childhood experiences on brain structure and function, neuroimaging studies identified through systematic searching were assessed for inclusion by two independent reviewers (GA, EL). Firstly, title and abstract screening was carried out, assessing against the predefined inclusion and exclusion criteria. Secondly, where it was not possible to determine eligibility from the title and abstract, full-text screening was performed. When discrepancies were raised, they were resolved through discussion, or, when they remained, by consultation with a third party (DS, LC). Cohen's kappa^43^ was computed to assess the agreement between two reviewers ‘include’, ‘maybe include’, or ‘exclude’ decisions during the full-text screening.

When the selection process was completed, data were extracted according to our predefined protocol. Data extracted from each study included publication details (e.g. authors, year of publication, country where the study was carried out) and demographic characteristics of the sample such as age and sex. Moreover, the clinical diagnosis was extracted for MDD and chronic pain along with type and scale of measurements for adverse childhood experiences, MDD, and chronic pain. The neuroimaging method used was also extracted along with the modality, the patient groups, use of contrast (if task-related functional MRI [fMRI]) and the resulting significant coordinates (*x*, *y*, *z* coordinates of the brain regions).

### Quality assessment

There is no standardised tool for assessing the risk of bias in MRI (structural and functional), to the best of our knowledge. Therefore, we modified a version of the Newcastle–Ottawa scale,^44^^,^^45^ which is used for assessing the quality of non-randomised studies, including case-control and cohort studies, in meta-analyses. The risk of bias tool included four categories, study selection aspects (adequate case definition: MDD, chronic pain, and adverse childhood experiences, representative of the cases, selection of controls and definition of controls), comparability (age, sex, and other variables), exposure (ascertain of exposure and drop-out rate), and statistical interference (uncorrected *P*-value threshold and correction for multiple testing at each voxel).

### Activation likelihood estimate meta-analysis method

A coordinate-based meta-analysis approach, activation likelihood estimate (ALE), of extracted neuroimaging results^46^, ^47^, ^48^ was used to analyse studies within BrainMap GingerALE 3.0.2 (http://www.brainmap.org/ale). Talairach space coordinates were converted into Montreal Neurological Institute (MNI) coordinates using the ‘icbm2tal’ transform.^49^ To take into account the spatial statistical uncertainty of the reported foci in the ALE method, foci were modelled as a 3D Gaussian probability distribution.^48^ Considering between-subject and between-template variance for each study, the full width half maximum (FWHM) of the Gaussian probability was computed.^46^ FWHM of the modelled probability was computed through combining the activation probabilities of the reported foci, for each analysed study.^46^ By combining the activation probabilities of the reported foci, modelled activation maps were computed for each analysed study. The union of these modelled activation maps as ALE scores were calculated within a grey matter on a voxel-by-voxel basis. ALE scores are tested against a null distribution of random spatial events to test for statistical significance.^46^

Cluster-level family-wise error (I) has greater sensitivity and specificity and is less prone to type one error with regard to the convergence in comparison with voxel-wise thresholding. In particular, the foci convergence is achieved over the random clustering of the foci-noise via testing against the null hypothesis of random spatial association between experiments.^46^ Thus, the I was used to guide the meta-analysis. Statistical significance was set at a corrected threshold of *P*<0.05 (using a threshold derived from the permutation of 1000 cluster-forming events assuming a voxel level of *P*<0.05). Computations involving the same group of subjects were pooled into one experiment to control for sample overlap.^48^^,^^50^ A random-effects analysis was used (GingerALE 3.0.2) with incorporation of variable uncertainty based on subject size,^46^ where the subject size is determined as the smallest number of participants in the contrast of the groups.^51^ We performed a meta-analysis across all fMRI and voxel-based morphology paradigms contrasting participants who experienced adverse childhood experiences with participants who did not experience adverse childhood experiences. In addition, where there was a sufficient number of relevant studies, we aimed to perform separate exploratory meta-analyses on MDD (or chronic pain) participants with adverse childhood experiences, MDD participants without adverse childhood experiences, separately for fMRI and VBM and fMRI MDD with adverse childhood experiences compared with healthy controls. An assessment of heterogeneity is frequently used in non-neuroimaging studies; however, there is not yet any accepted method to calculate this for coordinate-based meta-analyses. Therefore, we were unable to assess this formally.

## Results

After removing duplicates, 228 studies on mood disorder and adverse childhood experiences and 15 studies on chronic pain and adverse childhood experiences were identified, from title and abstract screening. After title, abstract, and – where appropriate – full-text screening, 43 studies were identified as meeting inclusion criteria for mood disorders and adverse childhood experiences, with six studies for chronic pain and adverse childhood experiences.^32^, ^33^, ^34^, ^35^, ^36^, ^37^, ^38^^,^^52^, ^53^, ^54^, ^55^, ^56^, ^57^, ^58^, ^59^, ^60^, ^61^, ^62^, ^63^, ^64^, ^65^, ^66^, ^67^, ^68^, ^69^, ^70^, ^71^, ^72^, ^73^, ^74^, ^75^, ^76^, ^77^, ^78^, ^79^, ^80^, ^81^, ^82^, ^83^, ^84^, ^85^, ^86^, ^87^, ^88^, ^89^, ^90^, ^91^, ^92^, ^93^ No studies were identified of adverse childhood experiences with comorbid mood disorder and chronic pain. Cohen's kappa was computed with good agreement between the two reviewers: kappa=0.69 (95% confidence interval [CI], 0.57–0.82; *P*<0.0001) for full-text study selection. Of the 108 full-text studies screened, 43 met inclusion criteria: 26 were structural MRI studies, seven were resting-state fMRI studies, and 10 were task-based fMRI studies (Supplementary material). Most of these studies were cohort studies or case-control studies. Details of the included structural studies can be found in Supplementary Table S2, and task-based fMRI studies can be found in Supplementary Table S3. Summary tables of imaging findings for the structural and task-based fMRI can be found in Table 1, Table 2, respectively. Table 3 summarises identified resting state fMRI studies: it was not possible to carry out ALE meta-analysis because of inadequately reported coordinates in those studies. The risk of bias is summarised in the Supplementary material. The assessment of study quality showed no study at high risk, nine studies at an intermediate risk of bias, and 40 studies at low risk of bias.

**Table 1:** Summary table of the main result for the structural MRI studies. adverse childhood experiences, adverse childhood experiences; BDI-II, Beck's Depression Inventory; CECA, Childhood Experience of Care and Abuse; CM, childhood maltreatment; CN, childhood neglect; CTQ, Childhood Trauma Questionnaire; CTQ-SF Childhood Trauma Questionnaire-Short Form; EA, emotional abuse; ELA, early life adversity; GMV, grey matter volume; HAD, Hospital Anxiety and Depression Scale; HAMD, Hamilton depression scale; HAMD-D, Hamilton Rating Scale for Depression; HDRS, Hamilton Depression Rating; HC, healthy control; IBS, irritable bowel syndrome; IDS-SR, Inventory of Depressive Symptomatology-Self report; LS, life stress; MADRS, Montgomery Åsberg Depression Rating Scale; MDD, major depressive disorder; PHQ, Patient Health Questionnaire; QIDS, Quick Inventory for Depression Symptomatology; SA, sexual abuse; SDS, Self-rating Depression Scale; ROD, recent onset of depression; SPD, somatoform pain disorder.

Authors	Structural MRI	
	Contrast of interest	Main results
***Studies examining the relationship of MDD or chronic pain with adverse childhood experiences and without adverse childhood experiences***		
Chaney and colleagues^53^	MDD+CM>MDD-CM	Compared with MDD without CM, MDD with CM: smaller hippocampal volume, larger dorsomedial prefrontal cortex, and orbitofrontal cortex.
Colle and colleagues^55^	MDD+ELA>MDD-ELA	MDD without ELA, compared with MDD with ELA: smaller hippocampal volumes found in males only.
Gerritsen and colleagues^35^	MDD+CM>MDD-CM	CM: no change in hippocampal volume. MDD: was associated with smaller hippocampal volume. MDD was related to smaller hippocampal volume, in participants with CM.
Monninger and colleagues^67^	LS	Reduce cortical thickness in the right medial orbitofrontal cortex and increased depressive symptoms were associated with chronic LS during infancy. Reduce cortical thickness in the left medial orbitofrontal cortex was associated with chronic LS during childhood and negatively correlated with the left caudal ACC and the left parahippocampal surface area. CT of the right transverse temporal lobe and the left entorhinal cortex volume was inversely related to LS during adolescence.
Oshri and colleagues^69^	adverse childhood experiences	Participants with higher adverse childhood experiences scores had smaller right amygdala volumes and smaller central-medial nuclei. Moreover, participants with higher adverse childhood experiences scores with increased anxiety, depressive symptoms and alcohol use showed smaller basolateral amygdala volume.
Peng and colleagues^70^	MDD-CN>MDD+CN	Compared with patients without CN, patients with CN showed increased WM densities in bilateral sublobar extra-nucleus and right brainstem midbrain
Salokangas and colleagues^72^	ROD+adverse childhood experiences >ROD-adverse childhood experiences	ROD was not found to be associated with changes in the amygdala–hippocampus complex in adulthood. ROD patients with experiences of physical abuse in early life showed that are mediating the reduction of frontal lobe.
Van Harmelen and colleagues^80^	CEM>NO CEM	Compared with non-CEM participants, CEM participants: reduction in bilateral dorsal medial prefrontal cortex, independent of comorbid psychopathology
Vythilingam and colleagues^81^	MDD+CA>MDD-CA	Compared with participants with MDD without CA and HC, participants with MDD with CA showed bilateral smaller hippocampal volumes.
Yang and colleagues^85^	MDD+CM>MDD-CM	For MDD with CM compared with MDD without CM: smaller left inferior occipital gyrus and larger left cerebellum anterior lobe and left superior temporal gyrus. Interaction effect for the CM and MDD for the MDD with CM compared with MDD without CM showed increased left superior frontal gyrus and right middle frontal gyrus and smaller right inferior frontal gyrus.
***Studies examining the different relationships of MDD, chronic pain, Healthy controls, and adverse childhood experiences***		
Chaney and colleagues^53^	MDD+CM>HC	Compared with HC, MDD with CM had smaller left orbitofrontal cortex and left dorsomedial prefrontal cortex.
Peng, and colleagues^70^	HC>MDD+CN	Compared with HC, MDD patients with CN showed decreased WM densities in bilateral inferior parietal lobules.
Yang and colleagues^85^	HC+CM>HC-CM	The main effect of CM had been observed for HC with CM compared with HC without CM, smaller left posterior cingulate cortex and larger right inferior frontal gyrus.

**Table 2:** Summary table of the main result for the functional MRI studies. adverse childhood experiences, adverse childhood experiences; BDI-II, Beck's Depression Inventory; CECA, Childhood Experience of Care and Abuse; CM, childhood maltreatment; CTQ, Childhood Trauma Questionnaire; CTQ-SF, Childhood Trauma Questionnaire-Short Form; EA, emotional abuse; ELA, early life adversity; ELT, early life trauma; GMV, grey matter volume; HAD, Hospital Anxiety and Depression Scale; HAMD, Hamilton depression scale; HAMD-D, Hamilton Rating Scale for Depression; HC, healthy control; HDRS, Hamilton Depression Rating; IBS, irritable bowel syndrome; IDS-SR, Inventory of Depressive Symptomatology-Self report; IP, internalizing psychopathology; MADRS, Montgomery Åsberg Depression Rating Scale; MDD, major depressive disorder; MSPD, multisomatoform pain disorder; PA, physical abuse; PHQ, Patient Health Questionnaire; QIDS, Quick Inventory for Depression Symptomatology; SA, sexual abuse; SDS, Self-rating Depression Scale; SPD, somatoform pain disorder. ∗Structural MRI provides detailed images of the brain's anatomy, whereas task-based functional MRI provides information about brain activity and function during a specific task.

Author	Task-based functional MRI∗			
	Task	Group of participants	Contrast of interest	Main results
Grant and colleagues^59^	Riksen flanker task of selective attention	MDD+ELA	Sad>neutral	In contrast to the sad > neutral faces, the right amygdala showed higher activation for the unipolar depressed patients with ELA. Increased activation of the amygdala was found, and a positive correlation with the PA. Other forms of abuse and neglect were also found to have a weaker relationship. The correlation was significant when the patient had experienced ELA, and it was not significant for depressed patients.
		MDD+ELA	positive>neutral	For positive > neutral faces, sexual abuse showed a correlation with amygdala response.
Hentze and colleagues^62^	Affective ToM task	MDD	Emotional focus	The contrast of emotional focus revealed a negative correlation between amygdala and MADRS scores (depression).
		CTQ	Perspective taking	Perspective taking revealed a positive correlation between amygdala activation and CTQ total scores.
Miller and colleagues^66^	Continuous performance task (CPT); Go/No-go	MDD+CM> MDD-CM	CM *vs* no CM	During working memory updating on CPT, patients with and without CM differed in the activation of the right dorsolateral prefrontal cortex.
Noll-Hussong and colleagues^88^	Empathy-for-pain paradigm	MSPD+abuse > MSPD–abuse	MSPD+abuse *vs* > MSPD–abuse	Abused patients in contrast to those with no experience of abuse showed an activation in the left lateral and medial superior frontal gyrus. No-abused patients in contrast with the abuse group showed an activation in the left hippocampus.
Peters and colleagues^71^	Face/shapes interaction	IP+ELA	Angry>Shapes	Cuneus showed increase activation in the angry > shapes contrast for IP+ELA.
		IP+ELA	Fearful > Shapes	Increased activation in the anterior and posterior cingulate, superior parietal, precuneus, cuneus, superior frontal and inferior temporal was demonstrated in the contrast of fearful > shapes for IP+ELA.
Ringel and colleagues^89^	Three sets of repeated 39 s rectal distension separated by a 39-s rest period	All abuse>All non-abuse	All abuse *vs* All non-abuse	Abused patients in contrast to non-abuse patients with IBS showed an activation in the left mid-cingulate cortex and left posterior cingulate cortex.
		IBS+abuse > ALL	IBS with abuse *vs* all	IBS patients with abuse in contrast with all others showed an activation in the left mid-cingulate cortex, bilateral posterior cingulate cortex and a de-activation in bilateral supra genual cingulate cortex.
Skokauskas and colleagues^74^	Emotional attention shifting task	MDD	Judgement of emotion minus judgement of geometry after emotional neutral stimuli	Decrease activation with the contrast judgement of emotion minus judgement of geometry after emotional neutral stimuli on patients with MDD on the fusiform gyrus
		MDD+SA	Judgement of emotion minus judgement of geometry after emotional negative stimuli	Increased activation with the contrast of judgement of emotion minus judgement of geometry after emotional negative stimuli on patients with MDD and experiences of SA on the left inferior parietal lobe.
Grant and colleagues^60^	Gender identification variant of the Eriksen flanker task of selective attention	MDD>HC	MDD>HC	Based on medial prefrontal cortex–amygdala connectivity within the MDD group, association between exposure to ELT with failure of inhibition was observed. Association between negative causal pathways from medial prefrontal cortex to amygdala with non-ELT MDD patients, even though a reduced dorsolateral prefrontal cortex input compared with HC.
Tozzi and colleagues^77^	Attentional cognitive emotional task	HC>MDD	HC>MDD	HC in contrast to MDD patients revealed an activation in the right middle frontal gyrus, right hippocampus, right precuneus and left lingual gyrus.
Tozzi and colleagues^78^	Valence recognition of emotional images.	MDD>HC	MDD>HC	MDD patients compared with HC during valence recognition of emotional images showed de-activation in the bilateral inferior frontal gyrus.

**Table 3:** Summary table of patient characteristics, and main result for resting state MRI studies. adverse childhood experiences, adverse childhood experiences; BDI-II, Beck's Depression Inventory; CECA, Childhood Experience of Care and Abuse; CM, childhood maltreatment; CN, childhood neglect; chronic pain, chronic pain; CTQ, Childhood Trauma Questionnaire; CTQ-SF, Childhood Trauma Questionnaire-Short Form; EA, emotional abuse; EAL, early adverse life; ELA, early life adversity; ELS, early life stress; GMV, grey matter volume; GUPI, Genitourinary Pain Index; HAD, Hospital Anxiety and Depression Scale; HAMD, Hamilton Depression Scale; HAMD-D, Hamilton Rating Scale for Depression; HC, healthy control; HDRS, Hamilton Depression Rating; IBS, irritable bowel syndrome; IDS-SR, Inventory of Depressive Symptomatology-Self report; MADRS, Montgomery Åsberg Depression Rating Scale; MDD, major depressive disorder; PHQ, Patient Health Questionnaire; QIDS, Quick Inventory for Depression Symptomatology; RSN, Resting State Network; SA, sexual abuse; SDS, Self-rating Depression Scale; SPD, somatoform pain disorder; UCPPS, urological chronic pelvic pain syndrome.

Author	Country	Study population			Type of study	Assessment of ELA	Assessment of chronic pain	Assessment of depression	Assessment of anxiety disorders	Resting-state functional MRI
		*n*	Mean age	Sex						Main results
Cisler and colleagues^54^	USA	HC=12 ELS-MDD=7 ELS+MDD=19	HC=25.92 (5.33) ELS-MDD=27.43 (7.39) ELS+MDD=31.28 (8.57)	–	An examination of global connectivity and hub-like properties in women with MDD with and without ELS compared with HC.	CTQ	n.a.	HAMD	n.a.	Between resilient individuals there were hub-like properties and decreased global connectivity for the right ventrolateral prefrontal cortex and for the dorsal anterior cingulate decreased local network connectivity. Between susceptible individuals there were hub-like properties and decreased global connectivity for the left amygdala and for the dorsal anterior cingulate decrease hub-like properties and for the left ventrolateral prefrontal cortex decreased local connectivity.
Wang and colleagues^82^	China	HC=20 MDD-CN=20 MDD+CN=18	HC=27.9 (4.4) MDD-CN=28.2 (8.7) MDD+CN=28.3 (6.2)	HC=11F MDD-CN=8F MDD+CN=10F	An investigation in MDD patients with and without CM of the whole-brain functional connectivity patterns.	CTQ	n.a.	HDRS Self-rating Depression Scale	n.a.	Compared with HC, MDD group in bilateral ventral medial prefrontal cortex/ventral anterior cingulate cortex revealed decreased functional connectivity strength. Compared with the MDD group without CM, MDD with CM in brain regions within the prefrontal–limbic–thalamic–cerebellar circuitry showed widespread reduction of functional connectivity strength, whereas the reductions were correlated with childhood neglect measurements.
Wu and colleagues^83^	China	HC=58 MDD=29	HC=27.9 (5.9) MDD=26.7 (6.0)	HC=34F MDD=17F	An examination of certain brain functional connectivity patterns and their relationship to certain affective temperaments. In addition, whether the FCs contribute to depressive symptoms.	CTQ	n.a.	Temperament Evaluation of Memphis (TEMPS) HDRS	Hamilton Anxiety Rating Scale (HAM-A)	Compared with HC, in MDD patients the covariation between the partial least square's functional connectivity profile and the partial least squares affective–temperament profile was enhanced. The somatisation symptom dimension was associated with the affective temperament modulated functional connectivity profile in MDD patients when there was adjusted for age, sex, duration of illness, age on set and HARS scores.
Xu and colleagues^84^	China	HC=17 MDD+CM=15 MDD-CM=14	HC=28.94 (5.92) MDD+CM=28.33 (5.81) MDD-CM=32.36 (6.23)	HC=7F MDD+CM=6F MDD-CM=5F	An investigation of brain functionality in MDD patients with CM experience via a resting-state fMRI.	CTQ	n.a.	HAMD-17	n.a.	In the prefrontal cortex there was an increased amplitude of low-frequency fluctuation and altered function connection which was associated with MDD patients with CM compared with MDD without CM. MDD patients with CM from patients without CM were differentiated by the left frontal middle gyrus.
Yu and colleagues^86^	USA	HC=39 MDD=189	HC=37.1 (14.7) MDD=37.3 (13.0)	HC=25F MDD=123F	An investigation in patients with MDD and healthy controls for the network connectivity differences within and between RSNs.	CTQ	n.a.	QIDS HAMD	Mood and anxiety symptom questionnaire anxious arousal (MASQ)	Compared with HC, MDD patients were characterised by a network model with abnormalities in the decrease within-network connectivity in the FPN, the dorsal attention network, and the cingulo-opercular network, task-positive RSNs. The second abnormality is an increase of within-network connectivity in the DMN and salience network, intrinsic networks. The last abnormality is an increase of within-network connectivity in the sensorimotor network and visual network, sensory networks. The history of childhood trauma and current symptoms in MDD patients were associated with a multivariate pattern of different within- and between-network connectivities, which involves the cingulo-opercular network, FPN, dorsal attention network, subcortical regions, ventral attention network, auditory network, visual network, and sensorimotor network
Gupta and colleagues^92^	USA	HC=58 IBS=110	–	HC=30F IBS=72F	An investigation in IBS patients compare with HC on the integrity of resting state networks, emotional/pain networks and default mode network related to EALs and sex.	Early adverse life trauma (ETI)	n.a.	HAD	n.a.	A positive correlation between left frontal parietal ICN-striatum connectivity and Early Adverse Life was demonstrated primarily in male participants. Female participants had a positive correlation with the connectivity of right putamen and right frontal parietal ICN. IBS patients showed negative correlations with right frontal parietal ICN – precentral gyrus connectivity and positive correlation with left parietal ICN – right superior parietal lobe connectivity.
Gupta and colleagues^93^	USA	HC=86 UCPPS=85	HC=37.9 (12.23) UCPPS=39.36 (12.8)	HC=59F UCPPS=56F	An investigation of the role of EAL's in the central processes of chronic pain.	Childhood Traumatic Early Adversity (CTES)	Baseline GUPI QoL score; Pain severity; Urinary Severity	n.a.	n.a.	Compared with HC, UCPPS showed lower centrality in the right anterior insular. Compared with males HC, males UCPPS showed lower centrality in the right anterior insular. Compared with females with UCPPS, males with UCPPS showed lower centrality in the left posterior cingulate, middle temporal gyrus, angular gyrus and superior temporal sulcus, although it had greater centrality in the anterior midcingulate cortex and precuneus. In females with UPPS an association was observed between higher reports of ELAs and greater centrality in the left precuneus and left anterior midcingulate cortex.

Structural MRI experiments, voxel-based morphology were split based upon disease status:

1. MDD participants exposed to adverse childhood experiences were compared with MDD participants not exposed to adverse childhood experiences (five experiments; 13 foci; 277 participants), which revealed eight significant clusters in the anterior, occipital, temporal, frontal lobe, and putamen and insula (further details in Table 4).

**Table 4:** Three groups of fMRI experiments and two groups of voxel based morphology significant meta-analysis results using GingerALE. ALE, Activation likelihood estimation; BA, Brodmann area; CP, chronic pain; ELA, early life adversity; Hem, Hemispheres; MDD, major depressive disorder; MNI, Montreal Neurological Institute coordinates. ∗ Broadman Area not applicable.

Cluster	Hem	Lobes	Brain regions	BA	MNI			ALE	*P*	*Z*
					x	y	z			
***fMRI meta-analysis results***										
**MDD with adverse childhood experiences (*n*=95) compared with HC (*n*=69)**^59^^,^^74^^,^^76^								164 participants		
1	Left	Parietal	Superior parietal lobule	7	–30	–42	48	0.0012	0.0180	2.09
	Left	Parietal	Inferior parietal lobule	40	–33	–49	49	0.0086	0.0003	3.41
2	Right	Limbic	Parahippocampal gyrus	30	21	–48	4	0.0009	0.0210	2.03
	Right	Limbic	Parahippocampal gyrus	30	12	–49	5	0.0090	0.0002	3.62
3	Left	Frontal	Middle frontal gyrus	9	–42	14	22	0.0095	2.3E–05	4.08
	Left	Frontal	Inferior frontal gyrus	9	–54	23	22	0.0092	6.1E–05	3.84
	Left	Frontal	Middle frontal gyrus	9	–45	29	31	0.0086	0.0003	3.41
4	Right	Frontal	Middle frontal gyrus	9	35	35	31	0.0051	0.0031	2.73
5	Left	Temporal	Middle temporal gyrus	37	–60	–52	–11	0.0092	6.1E–05	3.84
	Left	Temporal	Inferior temporal gyrus	37	–57	–46	–17	0.0089	0.0002	3.52
6	Right	Sublobar	Putamen/globus pallidus	∗	20	–4	10	0.0065	0.0014	2.99
**MDD with adverse childhood experiences (*n*=116) compared with MDD without adverse childhood experiences (*n*=154)**^59^^,^^66^^,^^71^^,^^74^								270 participants		
1	Right	Occipital	Middle occipital gyrus	18	29	–90	24	0.0180	1.1E–08	5.6
	Right	Occipital	Superior occipital gyrus	19	24	–90	38	0.0170	8.1E–08	5.24
	Right	Occipital	Cuneus	18	22	–81	28	0.0000	0.0830	1.38
	Right	Parietal	Precuneus	7	23	–61	45	0.0005	0.0440	1.71
2	Left	Parietal	Superior parietal lobule	40	–36	–54	51	0.0022	0.0160	2.15
	Left	Parietal	Inferior parietal lobule	40	–33	–40	49	0.0000	0.0800	1.4
**MDD or CP or HC: with adverse childhood experiences (*n*=139) > without adverse childhood experiences (*n*=217)**^66^^,^^71^^,^^74^^,^^88^^,^^89^								356 participants		
1	Left	Limbic	Posterior cingulate gyrus	31	–12	–34	42	0.0110	1.4E–05	4.18
		Parietal	Inferior parietal lobule	40	–36	–54	51	0.0022	0.0200	2.05
	Left	Parietal	Precuneus	7	–20	–46	48	0.0070	0.0018	2.92
	Left	Parietal	Inferior parietal lobule	40	–45	–34	49	0.0069	0.0018	2.91
***Structural (VBM) meta-analysis results***										
**MDD with adverse childhood experiences (*n*=154) compared with MDD without adverse childhood experiences (*n*=123)**^52^^,^^53^^,^^65^^,^^70^^,^^85^								277 participants		
1	Left	Anterior	Cerebellum	∗	–2	–58	–6	0.0093	2.5E–05	4.06
	Left	Occipital	Lingual gyrus	18	–8	–72	6	0.0090	3.2E–05	3.99
	Left	Anterior	Cerebellum	∗	–6	–50	–20	0.0089	0.0001	3.81
	Right	Anterior	Cerebellum	∗	8	–34	–14	0.0078	0.0004	3.37
2	Right	Temporal	Hippocampus	∗	32	–37	5	0.0075	0.0004	3.36
3	Right	Sub-lobar	Superior insula	∗	27	–13	20	0.0078	0.0004	3.37
4	Left	Sub-lobar	Putamen	∗	–25	–17	18	0.0078	0.0004	3.37
5	Left	Temporal	Superior temporal gyrus	41	–51	–33	14	0.0084	0.0001	3.71
6	Right	Temporal	Inferior temporal gyrus	20	46	–32	–21	0.0086	8.1E–05	3.77
7	Left	Frontal	Superior frontal gyrus	8	–17	32	37	0.0090	6.5E–05	3.83
8	Right	Frontal	Superior frontal gyrus	6	26	11	51	0.0090	6.5E–05	3.83
**MDD and HC with adverse childhood experiences (*n*=315) >MDD and HC without adverse childhood experiences (*n*=313)**^52^^,^^53^^,^^64^^,^^65^^,^^70^^,^^80^^,^^85^								628 participants		
1	Left	Occipital	Lingual gyrus	18	–8	–72	6	0.0180	1.7E–08	5.52
	Left	Anterior	Cerebellum	∗	–2	–58	–6	0.0093	0.0001	3.74
	Left	Anterior	Cerebellum	∗	–6	–50	–20	0.0089	0.0002	3.58
2. MDD participants and healthy subjects exposed to adverse childhood experiences were compared with MDD participants and healthy subjects not exposed to adverse childhood experiences (11 experiments; 26 foci; 628 participants), which revealed one significant cluster in the occipital and anterior lobe (further details in Table 4).
3. Chronic pain participants exposed to adverse childhood experiences compared with chronic pain participants not exposed to adverse childhood experiences: no studies were identified.

Task-based fMRI experiments were split based upon disease status:

1. MDD participants exposed to adverse childhood experiences compared with healthy subjects (five experiments; 18 foci; 164 participants) which revealed six significant clusters in the parietal, limbic, frontal, temporal lobe and putamen/globus pallidus (further details in Table 4).
2. MDD participants exposed to adverse childhood experiences compared with MDD participants not exposed adverse childhood experiences (seven experiments; 28 foci; 270 participants), which revealed two significant clusters in the occipital and parietal lobes (further details in Table 4).
3. MDD, or chronic pain participants or healthy subjects exposed to adverse childhood experiences compared with those not exposed to adverse childhood experiences (10 experiments; 37 foci; 296 participants), which revealed one significant cluster in the limbic and parietal lobe (further details in Table 4).
4. Chronic pain participants exposed to adverse childhood experiences compared with chronic pain participants not exposed to adverse childhood experiences (two experiments; nine foci; 23 participants) were not analysed because of the small group size.

## Discussion

**Fig 1:**
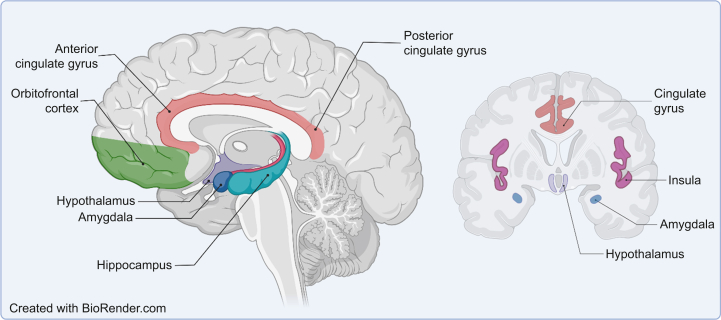
Key brain regions that are commonly associated with pain perception, including the insula, anterior and posterior cingulate, hypothalamus, hippocampus, amygdala, orbitofrontal cortex, and thalamus.

Adverse childhood experiences involving stress, injury, or diseases can change the brain at different levels, including epigenetic,^94^ cell biological, and systems and network levels.^95^^,^^96^ Using a systematic review and meta-analysis, we have explored adverse childhood experiences as a potential modulator for chronic pain and depression in adulthood. Significant structural alterations for patients with depression and adverse childhood experiences in the hippocampus, insula, and putamen were identified, with functional alteration in the precuneus. Investigation of the effects of adverse childhood experiences on chronic pain or depression revealed significant functional changes in the posterior cingulate cortex (PCC) and precuneus (Fig 1). We have shown that adverse childhood experiences have been linked with alterations in the function of the brain in people with chronic pain. Epigenetic and epidemiological studies have shown a relationship between adverse childhood experiences and chronic pain in later life^94^^,^^96^, ^97^, ^98^, ^99^, ^100^; however, we have identified a gap in the neuroimaging literature studying the long-term impact of adverse childhood experiences on neural processing in people with chronic pain. There are neuroimaging studies examining psychosocial adversities (e.g. physical, sexual, emotional abuse), but there are no studies examining non-psychosocial adversities such as those arising from serious medical conditions (e.g. asthma, cancer, epilepsy in early life) which have often been overlooked.^101^

The reward-motivation network (including the prefrontal cortex, nucleus accumbens, hippocampus, and ventral tegmentum) and the descending pain modulatory system (prefrontal cortex, anterior cingulate cortex [ACC], amygdala, hypothalamus) are implicated in vulnerability to painful conditions with evidence for structural, functional, and neurochemical alterations in the brain (Table 5).^102^ Other areas that might not affect risk of developing chronic pain, but are relevant to pain perception include changes in the insula, thalamus, orbitofrontal, primary and secondary somatosensory cortex, and dorsal ACC extending to mid-ACC.^102^^,^^103^ There is also an extensive literature around the involvement of the reward-motivation and salience networks in people with MDD.^104^^,^^105^ The neural correlates of emotional processing have been well studied in people who have experienced adverse childhood experiences, although reward processing has received less attention, but there is evidence that suggests a deficit in reward sensitivity.^106^^,^^107^

**Table 5:** Listing specific brain areas and their possible relation to pain, chronic pain, MDD and adverse childhood experiences. ACC, anterior cingulate cortex; adverse childhood experiences, adverse childhood experiences; MDD, major depressive disorder; PCC, posterior cingulate cortex.

Regions	Involvement of MDD, chronic pain, and adverse childhood experiences
Prefrontal cortex	Involvement in vulnerability to painful conditions (reward-motivation network and descending pain modulatory system).^102^ Involvement of the reward-motivation and salience networks in people with MDD.^104^^,^^105^ Deficit in reward sensitivity and reward anticipation in adverse childhood experiences.^106^^,^^107^
ACC	Individuals who are vulnerable to painful conditions may have changes in the reward-motivation and descending pain modulatory systems in the brain.^102^ These changes may not necessarily affect the risk of developing chronic pain, but they do play a role in how pain is perceived.^103^^,^^108^^,^^126^ Activated by other aversive experiences, leading to negative emotions and anxiety.^108^, ^109^, ^110^ MDD may have alterations in the reward-motivation and salience networks.^104^^,^^105^ adverse childhood experiences have been linked to a deficit in reward sensitivity and reward anticipation.^106^^,^^107^
Amygdala	Vulnerability to painful conditions may have changes in the descending pain modulatory system in the brain.^102^ They play a role in how pain is perceived.^103^ MDD have alterations in the reward-motivation and salience networks^104^^,^^105^ which can contribute to their vulnerability to painful conditions.
Hypothalamus/thalamus	Vulnerability to painful conditions (descending pain modulatory system),^102^ may not affect risk of developing chronic pain but is a relevant region to pain perception.^103^ Involvement with the MDD.^104^^,^^105^
Nucleus accumbens	Individuals who are at risk of experiencing painful conditions may have changes in the reward-motivation network in the brain.^102^ MDD may have alterations in the reward-motivation and salience networks,^104^^,^^105^ which can make them more susceptible to painful conditions. adverse childhood experiences have been linked to a deficit in reward sensitivity.^106^^,^^107^ Anhedonia, chronic pain, and depression is linked to dopamine abnormalities affected by inputs from the hippocampus. Research also suggests that childhood adversity can lead to dysfunction of the basal ganglia during reward anticipation.
Ventral tegmentum	Vulnerability to painful conditions (reward-motivation network).^102^ Involvement of the reward-motivation and salience networks in people with MDD.^104^^,^^105^ Deficit in reward sensitivity and dysfunction during reward anticipation in humans exposed to adverse childhood experiences.^106^^,^^107^
Hippocampus	Vulnerability to painful conditions (reward-motivation network), is a relevant region to pain perception.^103^ Involvement of the reward-motivation and salience networks in people with MDD.^104^^,^^105^ Children exposed to traumatic events have smaller hippocampus and weaker activity in that region during memory tasks.^116^ This is associated with difficulties in learning and memory in individuals with chronic pain.^130^, ^131^, ^132^ Changes in the structure of the hippocampus, which is often impacted by chronic pain, may contribute to the emergence of depression.^133^^,^^134^
Insula	It is an important area in regard to the perception of pain.^103^^,^^108^ It is activated by other negative experiences as well, leading to negative emotions and anxiety.^108^, ^109^, ^110^ Blunted signal in aversive stimuli in MDD. Mediates changes in the default mode network and frontoparietal network facilitating responses to salient stimuli.^108^^,^^115^ Increased insula activation in children exposed to violence.^116^, ^117^, ^118^, ^119^, ^120^, ^121^
Orbitofrontal cortex	It is still an area of the brain that plays a role in the perception of pain.^103^ MDD may have alterations in the reward-motivation and salience networks.^104^^,^^105^
Primary/secondary somatosensory cortex	A significant area in terms of pain.^103^
PCC	It is a significant area in terms of the sensation and perception of pain,^103^ and episodic memory retrieval.^122^ MDD have been found to have abnormal activity in this region, and healthy individuals who have experienced adverse childhood experiences. PCC activation may be more related to the emotional and memory-related aspects of stimuli, rather than the actual experience of pain.^122^

Our findings have demonstrated an impact of adverse childhood experiences on neuroanatomically plausible areas associated with mood and pain processing. Major cortical projections from the spinothalamic tracts (associated with pain) include the posterior insula, parietal operculum, and mid-cingulate cortex.^108^ Posterior insula and parietal operculum stimulation and focal seizures can trigger pain, lesions can cause pain deficits, and cortical insula damage has been correlated with neuropathic pain.^108^ Other regions, including anterior insula and anterior cingulate, influence the experience of pain but are also activated by other aversive experiences generating negative affect and anxiety.^108^, ^109^, ^110^ Lesions in the posterior insula^111^ and mid-cingulate cortex^112^ can result in patients continuing to recognise pain but with reduced or absent suffering; pain asymbolia.^113^ The anterior insula is thought to alter responsiveness to specific stimuli^114^ because of its involvement in the salience network,^108^ which mediates changes in the default mode network and frontoparietal network facilitating responses to salient stimuli.^115^ Notably, a systematic review on childhood adversity and neural development highlighted the importance of the salience network,^116^ with increased insula activation in children exposed to violence.^117^, ^118^, ^119^, ^120^, ^121^

A meta-analysis of functional imaging studies reported that activation of the PCC was associated with the experience of pain and episodic memory retrieval^122^ consistent with earlier reports.^123^, ^124^, ^125^ The anterior mid-cingulate cortex (aMCC) is also strongly associated with the experience of pain,^108^^,^^126^ and for some patients, small lesions of the aMCC relieve the distress of intractable pain.^112^ Relative to the aMCC though, a larger and more posterior part of the cingulate is also associated with pain.^122^ Furthermore, there is evidence for a rostral–caudal segregation of function, with the anterior PCC region more associated with pain and the posterior PCC region more associated with memory.^122^ PCC activation appears consistently associated with the emotional salience of stimuli and, despite evidence for segregation, there is a tendency for emotion and memory-related activations to overlap.^122^ Our meta-analysis study is consistent with these reports, such that patients with chronic pain and MDD were found to have reproducibly abnormal PCC activity. Furthermore, it is worth noting that healthy subjects with a history of adverse childhood experiences also have abnormal PCC activation. Consequently, abnormal activation of this region may be associated more with emotion and memory-related activations of the PCC rather than experiences of pain.

The hippocampus has an important role in the storage and retrieval of long-term explicit memories^127^ and in associative learning.^128^ It also has an important role in terminating the stress response via its regulatory role in the hypothalamic–pituitary–adrenal axis.^129^ Hippocampal volume and functional abnormalities have been reported inconsistently in deprivation-exposed children.^116^ Nevertheless, in children exposed to threat-related adversity, reduced hippocampal volumes have been reported, consistent with our findings including reduced activation during a memory task.^116^ Reduced hippocampal volume has been reported in patients with chronic pain: fibromyalgia,^130^ complex regional pain syndrome (CRPS), and chronic back pain (CBP),^131^ with evidence for learning and memory deficits in patients with chronic pain.^132^ Chronic pain and depression comorbidity is common, and it has been suggested that hippocampal abnormalities observed in patients with chronic pain may be related to the mood component.^133^ Anhedonia, chronic pain, and depression have all been correlated with blunted striatum activation which may be related to dopaminergic abnormalities modulated by hippocampal afferents.^134^ Studies on animals and humans have revealed reduced neurogenesis, neuroplasticity, neurotropic factors and increased hippocampal inflammation in both patients with depression and chronic pain.^132^

The reward system includes the caudate and putamen,^135^ with these regions responding to both the receipt and anticipation of reward.^136^ Consistent with anhedonia being a prominent clinical feature of depression, the brain reward circuitry appears abnormal in depression with functional dysregulations and brain structure changes,^137^ in accordance with our findings, such as blunted striatal responses to anticipated reward in maltreated children.^106^^,^^107^ Blunted reward-linked striatal activity has also been observed in patients with adverse postnatal experiences.^138^ Dysfunction of the basal ganglia during reward anticipation in humans exposed to childhood adversity may reflect abnormalities in dopaminergic circuits reported in animal studies.^139^^,^^140^

### Limitations

Potential limitations of this study should be noted. First, an assessment of heterogeneity is often done in non-neuroimaging studies, but there is no methodology for assessing heterogeneity for ALE or other coordinate-based meta-analyses.^141^ The ALE method provides an estimation of the probability that an activity in a specific region may differ between groups of patients and not an estimate of the mean difference in the regional signal change.^142^ Thus, traditional measures of heterogeneity are not applicable.^141^^,^^143^^,^^144^ Second, the limited number of neuroimaging studies available in the literature investigating chronic pain with adverse childhood experiences limited the meta-analysis results. Third, adverse childhood experiences have been associated with other psychiatric disorders, such as post-traumatic stress disorder (PTSD) and anxiety, whereas our study focused only on patients with depression. Recall bias,^145^ unfortunately, cannot be eliminated because of the systematic error that occurs when the participants did not remember previous adverse childhood experiences accurately when answering the questionnaire. Prospective longitudinal cohort studies and pre-existing datasets that include information collected during childhood, which can be further linked to healthcare records in later life (e.g. Generation Scotland: STRADL-Pain,^146^, ^147^, ^148^ ALSPAC^149^), are approaches to address this limitation.

### Conclusions

This review has investigated the neural correlates of adverse childhood experiences on chronic pain and depression. It is notable that there are few neuroimaging investigations into patients with chronic pain who have experienced adverse childhood experiences. Nevertheless, significant brain structure and function correlations with adverse childhood experiences were observed in people with major depressive disorder or chronic pain in comparison with healthy controls. In the former, correlations were found in the hippocampus, superior insula, putamen, and precuneus, and in the latter, significant correlations were found in the dorsal anterior cingulate and precuneus. Our results indicate the existence of brain structural and functional abnormalities associated with adverse childhood experiences – some of which may be characteristic for adverse childhood experiences and others more related to comorbid depression and chronic pain. Moreover, they are suggesting a shared neural correlates for comorbidity and possibly increasing the vulnerability to develop later in life depression, chronic pain, or both.

## Authors’ contributions

Concept: GA, EL, LC, JDS

Study design: GA, EL, LC, JDS

Interpretation of data: GA, EL, LC, JDS

Drafting the manuscript: GA, EL, LC, JDS

Article screening and selection: GA, EL

Data extraction, analysis, and quality assessment of studies: GA

Read and approved the final version of the manuscript: all authors

## Acknowledgements

We are grateful to Research Librarian, Scott McGregor at Library and Learning and Culture and Information, University of Dundee for his kind assistance with design of the database searches. LC and JDS are part of the UKRI Advanced Pain Discovery Platform.

## Declarations of interest

LC is a member of the *British Journal of Anaesthesia* and *BJA Open* editorial boards. GA, EL, and JDS have no conflicts of interests to declare.

## Funding

The study is part of E-PaiD PhD project, funded by TENOVUS Scotland Research PhD Studentship, ref T20-18.
