# Supplementary material for: The effect of adverse childhood experiences on chronic pain and major depression in adulthood: a systematic review and meta-analysis
Source: Br J Anaesth. 2023 Apr 21;130(6):729–46. doi: 10.1016/j.bja.2023.03.008 (PMC10251130; doi:10.1016/j.bja.2023.03.008)
Supplement: Multimedia component 1 [file mmc1.docx]

# Supplementary material

| Search strategy | | | |
| --- | --- | --- | --- |
| 1 | Adverse Childhood Experiences | MeSH |  |
| 2 | ACEs |  |  |
| 3 | Adverse Childhood Exposure$ |  |  |
| 4 | Adversity child$ |  |  |
| 5 | Early life adversity |  |  |
| 6 | Child maltreatment |  |  |
| 7 | Child trauma |  |  |
| 8 | Child misfortune |  |  |
| 9 | 1 OR 2 OR 3 OR 4 OR 5 OR 6 OR 7 OR 8 |  |  |
| 10 | Chronic Pain | MeSH |  |
| 11 | Arthralgia | MeSH |  |
| 12 | Neuralgia | MeSH |  |
| 13 | Fatigue Syndrome, Chronic | MeSH |  |
| 14 | Fibromyalgia | MeSH |  |
| 15 | Widespread Chronic Pain |  |  |
| 16 | Nerve Pain |  |  |
| 17 | Neuropathic Pain |  |  |
| 18 | Chronic Fatigue Disorder |  |  |
| 19 | Chronic Fatigue-Fibromyalgia Syndrome |  |  |
| 20 | Fatigue Syndrome, Postviral |  |  |
| 21 | Diffuse Myofascial Pain Syndrome |  |  |
| 22 | Fibromyalgia-Fibromyositis Syndrome |  |  |
| 23 | Neuropathic |  |  |
| 24 | Rheumatism Muscular |  |  |
| 25 | 10 OR 11 OR 12 OR 13 OR 14 OR 15 OR 16 OR 17 OR 18 OR 19 OR 20 OR 21 OR 22 OR 23 OR 24 | | |
| 26 | Magnetic Resonance Imaging | MeSH |  |
| 27 | Functional Neuroimaging | MeSH |  |
| 28 | Neuroimaging | MeSH |  |
| 29 | Functional Magnetic Resonance Imaging |  |  |
| 30 | Brain Mapping |  |  |
| 31 | MRI |  |  |
| 32 | fMRI |  |  |
| 33 | f-MRI |  |  |
| 34 | Brain Imaging |  |  |
| 35 | Functional MRI |  |  |
| 36 | Functional Brain Imaging |  |  |
| 37 | 26 OR 27 OR 28 OR 29 OR 30 OR 31 OR 32 OR 33 OR 34 OR 35 OR 36 | | |
| 38 | Depression | MeSH |  |
| 39 | Depressive |  |  |
| 40 | Major Depressive Disorder |  |  |
| 41 | Phobi$ |  |  |
| 42 | Claustrophobia |  |  |
| 43 | Anxiety | MeSH |  |
| 44 | Angst |  |  |
| 45 | Anxiousness |  |  |
| 46 | Hypervigilance |  |  |
| 47 | Nervousness |  |  |
| 48 | MDD |  |  |
| 49 | MDS |  |  |
| 50 | Affective Disorder$ |  |  |
| 51 | Affective Phycho$ |  |  |
| 52 | Melancholia |  |  |
| 53 | Paraphrenia |  |  |
| 54 | Psychosis |  |  |
| 55 | Depotentiation |  |  |
| 56 | Seasonal Mood Disorder |  |  |
| 57 | Psychotic Mood Disorders |  |  |
| 58 | Dysthymic Disorder | MeSH |  |
| 59 | Dysthymia |  |  |
| 60 | Bipolar Disorder | MeSH |  |
| 61 | 38 OR 39 OR 40 OR 41 OR 42 OR 43 OR 44 OR 45 OR 46 OR 47 OR 48 OR 49 OR 50 OR 51 OR 52 OR 53 OR 54 OR 55 OR 56 OR 57 OR 58 OR 59 OR 60 | | |
| 62 | #9 AND #25 AND #37 | | |
| 63 | #9 AND #37 AND #61 | | |
| 64 | #9 AND #25 AND #37 AND 61 | | |
| 65 | #25 AND #37 AND #61 | | |

Suppl. Table 1 Search strategy


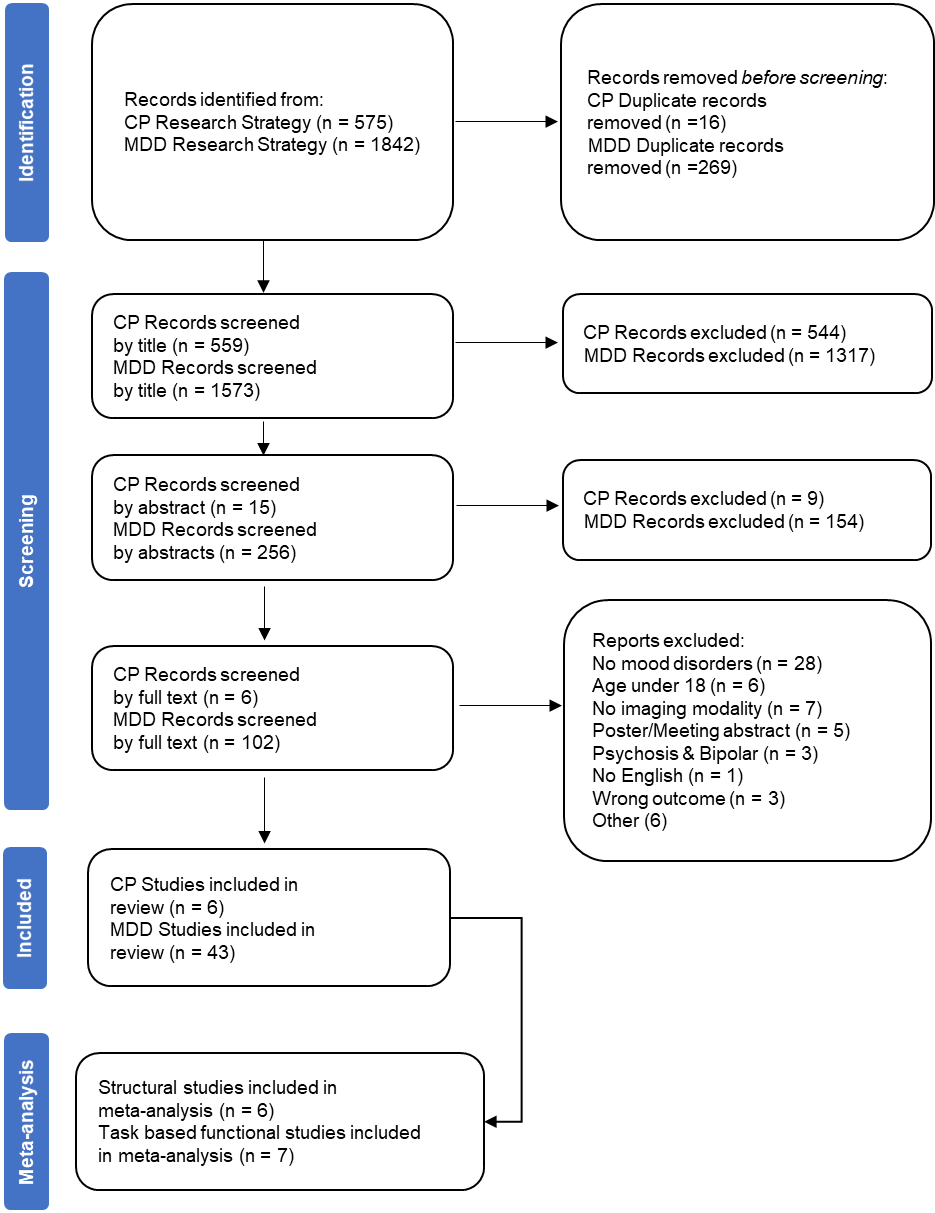


*Figure 1 Prisma flow chart of selection process for Systematic Review. MDD, Major Depressive Disorder; CP, Chronic pain.*

| **Author (year)** | **Country** | **Study population** | | | **Type of study** | **Assessment of ELA** | **Assessment of chronic pain** | **Assessment of depression** | **Assessment of anxiety** |  |  |
| --- | --- | --- | --- | --- | --- | --- | --- | --- | --- | --- | --- |
|  |  | **N** | **Sex** | **Mean (SD)  age (years)** |  |  |  |  |  |  |  |
| **Structural MRI studies** | | | | | | | | | |  |  |
|  |  |  |  |  |  |  |  |  |  |  | |
| **Ahn and colleagues^8*^** | USA | HC=26 | HC=19F | HC=31.4 (7.6) | MDD patients compared with HC assessing the experience of maltreatment during childhood. | Korean-CAEQ | n.a | Korean – QIDS | n.a |  |  |
|  |  | MDD+CA=23 | MDD+CA=20F |  |  |  |  |  |  |  |  |
|  |  | MDD-CA=11 | MDD-CA=9F | MDD-CA=32.7 (8.5) |  |  |  |  |  |  |  |
| **Carballedo and colleagues ^1^** | Republic of Ireland & Germany | HC= 71 | HC= 44F | HC=38.4 (13.5) | MDD patients compared with HC assessing the ELA on hippocampal volume | CTQ | n.a | BDI‐II | n.a |  |  |
|  |  | MDD= 62 | MDD= 38F | MDD=41.8 (11.1) |  |  |  | HAMD |  |  |  |
| **Chaney and colleagues^9*^** | Republic of Ireland | HC-CM=36 | HC-CM=24f | HC-CM=34.2 (10.8) | MDD patients with and without CM compared with HC with and without CM accessing brain structure. | CTQ-SF | n.a | HAM-D | n.a |  |  |
|  |  | HC+CM=10 | HC+CM=4f | HC+CM=45.3 (15.8) |  |  |  |  |  |  |  |
|  |  | MDD+CM=20 | MDD+CM=9f | MDD+CM=39.9 (9.7) |  |  |  | BDI-II |  |  |  |
|  |  | MDD-CM=17 | MDD-CM=12f | MDD-CM=40.6 (10.4) |  |  |  |  |  |  |  |
| **Colle and colleagues^11^** | France | MDD+ELA=28 | MDD+ELA=19F | MDD+ELA=43.6 (12.4) | MDD patients with and without ELA assessing hippocampal volume. | Assessed by two psychiatrists using patient health records. | n.a | HDRS-17 | n.a |  |  |
|  |  | MDD-ELA=35 | MDD-ELA=18F | MDD-ELA=48.5 (12.1) |  |  |  |  |  |  |  |
| **Frodl and colleagues (2010) Neuropsychopharmacology ^2^** | Germany | HC= 27 | HC= 18F | HC=41.9 (13.2) | MDD patients with ELA compared to HC assessing brain structure. | CTQ | n.a | HDRS-21 | n.a |  |  |
|  |  | MDD= 24 | MDD= 13F | MDD=43.8 (11.9) |  |  |  |  |  |  |  |
| **Frodl and colleagues (2010). J Psychiatr Res ^3^** | Ireland | HC= 44 | HC= 26F | HC=41.1 (12.5) | MDD patients with ELA compared to HC assessing brain structure. | CTQ | n.a | HDRS-21 | n.a |  |  |
|  |  | MDD= 43 | MDD= 42F | MDD=44.2 (12.2) |  |  |  |  |  |  |  |
| **Frodl and colleagues (2014) ^12^** | Ireland | HC= 44 | HC= 27F | HC=36.2 (13.3) | MDD patients with ELA compared to HC assessing brain structure. | CTQ | n.a | BDI‐II | n.a |  |  |
|  |  | MDD= 38 | MDD= 25F | MDD=40.9 (10.8) |  |  |  | HAMD |  |  |  |
| **Frodl and colleagues (2017) ^13^** | ENIGMA | HC= 2078 | HC= 944F | HC=46.3 (15.2) | MDD patients with ELA compared to HC assessing brain subcortical structures. | CTQ-SF | n.a | BDI-II | n.a |  |  |
|  |  | MDD= 958 | MDD= 614F | MDD=42.4 (14.3) |  |  |  | HDRS-17 |  |  |  |
|  |  |  |  |  |  |  |  | IDS-SR |  |  |  |
| **Gerritsen and colleagues ^4^** | Netherland | NESDA= 262 | NESDA= 226F | NESDA= 37.8 (10.2) | MDD patients with and without ELA assessing hippocampal volume. | Nemesis Trauma Interview | n.a | n.a | n.a |  |  |
|  |  | SMART= 636 | SMART= 55F | SMART= 61.5 (9.6) |  |  |  |  |  |  |  |
| **Gupta and colleagues (2016) ^47^** | USA | HC=137 | HC=137F | HC=28.62 (9.06) | IBS compared to HC evaluating the association of EALs and candidate gene polymorphisms in affecting the thickness of the subgenual anterior cingulate cortex. | Early Traumatic Inventory- | IBS | HAD | n.a |  |  |
|  |  | IBS=73 | IBS=73F | IBS=30.18 (9.43) |  | Self-Report (ETI-SR) |  |  |  |  |  |
| **Jaworska and colleagues ^19^** | Canada | HC=18 | HC=10f | HC=31.9(9.2) | MDD patients with and without CM compared with HC accessing brain cortical thickness. | CTQ-SF | n.a | HAMD-17 | n.a |  |  |
|  |  | MDD overall=36 | MDD overall=22f | MDD overall=37.1(11.2) |  |  |  |  |  |  |  |
|  |  | paediatric MDD=20 | paediatric MDD=12f | paediatric MDD=31.5(10.5) |  |  |  |  |  |  |  |
|  |  | adult MDD onset=16 | adult MDD onset=10f | adult MDD onset=44.1(7.7) |  |  |  |  |  |  |  |
|  |  | non-abuse=19 | non-abuse=10f | non-abuse=36.4(12.6) |  |  |  |  |  |  |  |
|  |  | abuse=12 | abuse=8f | abuse= 40.0(9.5) |  |  |  |  |  |  |  |
| **Lenze and colleagues ^5^** | USA | HC= 24 | HC= 24F | HC= 46(14) | MDD patients with and without CA assessing hippocampal volume. | CECA | n.a | HAMD-17 | n.a |  |  |
|  |  | Remitted MDD= 31 | Remitted MDD= 31F | Remitted MDD= 50(15) |  |  |  |  |  |  |  |
| **Lu, S and colleagues ^20*^** | China | MDD+CTE=16 | MDD+CTE=5F | MDD+CTE=24.4 (4.79) | MDD patients with and without childhood trauma exposures assessing GMV. | CTQ | n.a | Zung’s SDS | n.a |  |  |
|  |  | MDD-CTE=14 | MDD-CTE=8F | MDD-CTE=23.5 (5.77) |  |  |  |  |  |  |  |
|  |  | HC+CTE=24 | HC+CTE=15F | HC+CTE=21.5 (3.98) |  |  |  | HAMD-24 |  |  |  |
|  |  | HC-CTE=24 | HC-CTE=15F | HC-CTE=21.5 (3.69) |  |  |  |  |  |  |  |
| **Lu, X. and colleagues ^21*^** | China | MDD+CM=48 | MDD+CM=28F | MDD+CM=33.2(8.7) | MDD and HC with and without childhood trauma exposures assessing brain volume. | CTQ | n.a | HAMD-24 | n.a |  |  |
|  |  | MDD-CM=28 | MDD-CM=16F | MDD-CM=36.1(7.8) |  |  |  |  |  |  |  |
|  |  | HC+CM=48 | HC+CM=24F | HC+CM=33.0(7.8) |  |  |  |  |  |  |  |
|  |  | HC-CM=38 | HC-CM=19F | HC-CM=33.8(7.4) |  |  |  |  |  |  |  |
| **Meyer and colleagues (2020) ^46^** | Germany | HC=13 | HC=11F | HC=49.1(11.0) | SPD patients compare to HC assessing the interrelationship of exposure to childhood maltreatment and insular cortical thickness. | CTQ | Somatoform Pain Disorder | PHQ-15 | n.a |  |  |
|  |  | SPD=13 | SPD=9F | SPD=43.0(12.2) |  | German version |  | PHQ-9 |  |  |  |
| **Monninger and colleagues ^23^** | Germany | HC=190 | HC=107 | n.a |  | Munich Events List (MEL) | n.a | BDI | n.a |  |  |
|  |  | Participants=30 |  |  |  |  |  |  |  |  |  |
| **Opel and colleagues ^6^** | Germany | HC=85 | HC=51F | HC=37.2(11.8) | MDD patients compared with HC assessing the ELA on hippocampal volume | CTQ | n.a | HAM-D | n.a |  |  |
|  |  | MDD=85 | MDD=54F | MDD=37.6(12.0) |  |  |  | BDI |  |  |  |
| **Oshri and colleagues^25^** | USA | HC+MDD = 119 | HC+MDD = 119 | HC+MDD= 24.64(0.67) 62 WOMEN | Assess the effect of ACEs on amygdala morphometry and current psychiatric symptoms. | ACEs | n.a | PHQ-9 | General Anxiety Disorder −7 |  |  |
| **Peng, and colleagues ^26*^** | China | MDD+CN=19 | - | - | MDD patients with and without childhood neglect assessing white-matter density abnormalities. | CTQ | n.a | HAMD-17 Zung’s SDS | Dysfunctional Attitude Scale (DAS) |  |  |
|  |  | MDD-CN=21 |  |  |  |  |  |  |  |  |  |
|  |  | HC=20 |  |  |  |  |  |  |  |  |  |
| **Saleh and colleagues ^7^** | USA | HC=65 | HC=43F | HC=29.7 (9.2) | MDD patients compared with HC assessing the effect of ELS in brain morphology. | Early Life Stress Questionnaire (ELSQ) | n.a | HDRS-17 | n.a |  |  |
|  |  | MDD=64 | MDD=39F | MDD=35.1 (8.9) |  |  |  |  |  |  |  |
| **Salokangas and colleagues ^28^** | Europe | ROP=127 | - | - | Examine association between ROD and amygdala-hippocampus volume and mediate the effect of CAE on social anxiety and depression. | CTQ | n.a | BDI-II | Social Phobia Inventory (SPIN) |  |  |
|  |  | CHP=119 |  |  |  |  |  |  |  |  |  |
|  |  | ROD=128 |  |  |  |  |  |  |  |  |  |
| **Tozzi and colleagues (2020) ^34^** | ENIGMA | HC=2588 | HC=1303F | HC=43.3(15.9) | MDD patients compared with HC assessing the effect of CM in brain cortical thickness. | CTQ | n.a | HDRS-17 | n.a |  |  |
|  |  | MDD=1284 | MDD=813F | MDD=40.9(14.6) |  |  |  | BDI-II |  |  |  |
|  |  |  |  |  |  |  |  | IDS-SR |  |  |  |
| **Van Harmelen and colleagues ^36*^** | Netherland | No-CEM=97 | No-CEM=65F | No-CEM=36.57 (1.09) | CEM participants compare to no-CEM assessing the structural brain changes. | List of Threatening Events Questionnaire (LTE-Q) | n.a | MADRS | Beck Anxiety Inventory (BAI) |  |  |
|  |  | CEM=84 | CEM=55F | CEM=38.68 (1.09) |  |  |  |  |  |  |  |
| **Vythilingam and colleagues ^37^** | USA | MDD+CA=21 | MDD+CA=21 | MDD+CA=33(6) | MDD patients compared with HC assessing the effect of CA and/or PA in the hippocampus, temporal lobe, and whole brain | clinician-rated Early Trauma Inventory | n.a | HDRS-17 | Clinician-Administered PTSD Scale |  |  |
|  |  | MDD-CA=11 | MDD-CA=11 | MDD-CA=34(8) |  |  |  | Zung’s SDS |  |  |  |
|  |  | HC-CA=14 | HC-CA=14 | HC-CA=27(5) |  |  |  |  |  |  |  |
| **Yang and colleagues ^41*^** | China | MDD+CM=41 | MDD+CM=30F | MDD+CM=31.7 (8.0) | MDD patients compare to HC assessing the effect of CM in the grey matter volumes. | CTQ | n.a | HDRS-17 | n.a |  |  |
|  |  | MDD-CM=43 | MDD-CM=31F | MDD-CM=30.1 (7.5) |  |  |  |  |  |  |  |
|  |  | HC+CM=16 | HC+CM=12F | HC+CM=32.9 (7.6) |  |  |  |  |  |  |  |
|  |  | HC-CM=68 | HC-CM=49F | HC-CM=29.8 (6.5) |  |  |  |  |  |  |  |
| **Yuan and colleagues^43^** | USA | HC=44 | HC=26f | HC=33.3(11.5) | MDD patients compare to HC assessing the effect of CA in the subcortical volumes. | Yes/No questions “Any history of physical and/or sexual abuse over your lifetime?” and “If yes, did the abuse take place before 15 years of age?” | n.a | HAMD-17 | n.a |  |  |
|  |  | MDD=41 | MDD=24f | MDD=34.8(10.8) |  |  |  |  |  |  |  |
|  |  | MDD+CA=17 | MDD+CA=13f | MDD+CA=37.1(10.8) |  |  |  |  |  |  |  |
|  |  | MDD-CA=24 | MDD-CA=11f | MDD-CA=33.2(10.6) |  |  |  |  |  |  |  |
|  |  | CAMI:MDD=31 | CAMI:MDD=40.42 (9.72) | CAMI:MDD=21F |  |  |  |  |  |  |  |
|  |  |  | TCIN:HC=34.00 (11.63) |  |  |  |  |  |  |  |  |
|  |  | TCIN:HC=21 | TCIN:MDD=37.76 (13.17) | TCIN:HC=13F |  |  |  | BDI |  |  |  |
|  |  | TCIN:MDD=25 |  | TCIN:MDD=15F |  |  |  |  |  |  |  |

Suppl. Table 2 Summary Table, demographics for the structural MRI studies. *, studies included in the meta-analysis; MDD, Major Depressive Disorder; CP, chronic pain; HC, Healthy Control; ELA, Early Life Adversity; CM, Childhood Maltreatment; CTE, Childhood Trauma Exposure; EAL, Early Adverse Life; CN, Childhood Neglect; CA, Childhood Abuse; CEM, Childhood Emotional Maltreatment; SA, Sexual Abuse; EA, Emotional Abuse; CECA, Childhood Experience of Care and Abuse; CTQ, Childhood Trauma Questionnaire; CTQ-SF, Childhood Trauma Questionnaire-Short Form; ACEs, Adverse Childhood Experiences; BDI‐II, Beck’s Depression Inventory; QIDS , Quick inventory for depression symptomatology; HAMD, Hamilton depression scale; HAMD-D, Hamilton Rating Scale for Depression; HDRS, Hamilton Depression Rating; MADRS, Montgomery Åsberg Depression Rating Scale (MADRS); IDS-SR, Inventory of Depressive Symptomatology-Self report; HAD, Hospital Anxiety and Depression Scale; SDS, Self-rating Depression Scale; PHQ, Patient Health Questionnaire ; NESDA, Netherlands Study of Depression and Anxiety; GMV, Grey matter volume; IBS, Irritable bowel syndrome; CH, C-homozygous; TA T-allele; ROD, Recent onset Depression; CD, Chronic Depression; ROP, Recent onset Psychosis; CHP, Clinical High-risk to Psychosis; IP, Internalizing Psychopathology; SPD, Somatoform pain disorder; ToM, Theory of Mind; MSPD, Multisomatoform pain disorder; Eriksen flanker task, an fMRI task that allows for testing of both voluntary and nonvoluntary attention

| **Author (year)** | **Country** | **Study population** | | | **Type of study** | **Assessment of ELA** | **Assessment of chronic pain** | **Assessment of depression** | **Assessment of anxiety** |  |  |
| --- | --- | --- | --- | --- | --- | --- | --- | --- | --- | --- | --- |
|  |  | **N** | **Sex** | **Mean (SD)  age (years)** |  |  |  |  |  |  |  |
| **Task-based fMRI studies** | | | | | | | | | |  | |
|  |  |  |  |  |  |  |  |  |  |  | |
| **Grant and colleagues (2011) ^15*^** | USA | HC=16 | HC=31.1(9.2) | HC=10F | MDD patient’s examination of the experience of maltreatment during childhood via an Eriksen flanker task of selective attention on amygdala. | CTQ-SF | n.a | HDRS | n.a |  |  |
|  |  | MDD=2O | MDD=34.5(10.7) | MDD=11F |  |  |  |  |  |  |  |
|  |  | MDD-CM=10 | MDD-CM=29.2(9.3) | MDD-CM=7F |  |  |  |  |  |  |  |
|  |  | MDD+CM=10 | MDD+CM=39.3(9.5) | MDD+CM=4F |  |  |  |  |  |  |  |
| **Grant and colleagues (2014) ^16^** | USA | HC=19 | HC=31.2(9.2) | HC=10F | An evaluation of unmediated MDD and HC in task-based connectivity between medial or lateral prefrontal cortex and amygdala. | CTQ-SF | n.a | HDRS | Beck Anxiety Inventory (BAI) |  |  |
|  |  | MDD=20 | MDD=34.5(10.7) | MDD=11F |  |  |  |  |  |  |  |
|  |  | MDD-ELT=10 | MDD-ELT=29.2(9.3) | MDD-ELT=7F |  |  |  |  |  |  |  |
|  |  | MDD+ELT=10 | MDD+ELT=39.3(9.5) | MDD+ELT=4F |  |  |  |  |  |  |  |
| **Hentze and colleagues^18^** | Germany | CD=25 | CD=41.52 | CD=16F | MDD patient’s examination of the experience of maltreatment during childhood via an affective ToM task. | CTQ-SF | n.a | MADRS | n.a |  |  |
|  |  |  |  |  |  |  |  |  |  |  |  |
| **Miller and colleagues ^22*^** | Australia | MDD+CM=43 | MDD+CM=34.0(13.7) | MDD+CM=27F | MDD patient’s assessment of the contribution of the association between childhood maltreatment and antidepressant medication. | Early Life Stress Questionnaire (ELSQ) | n.a | MADRS | n.a |  |  |
|  |  | MDD-CM=65 | MDD-CM=33.3(12.6) | MDD-CM=20F |  |  |  |  |  |  |  |
| **Noll-Hussong and colleagues ^44*^** | Germany | HC=8 | HC=44.75 (22–61) | HC=7F | An investigation of multisomatoform pain patients with and without history of sexual abuse during early life of the neural substrates of empathy induced. | CTQ-34 | n.a | BDI | Spielberger’s State–Trait Anxiety Inventory (STAI-G) |  |  |
|  |  | MSPD=8 | MSPD=46.1 (22–67) | MSPD=7F |  |  |  |  |  |  |  |
| **Peters and colleagues^27*^** | USA | HC=30 | HC=25(9.98) | HC=15F | Assessment of impact of ELA in internalizing psychopathology, cortical brain activation to emotional faces. | Childhood Trauma Questionnaire (CTQ) | n.a | DASS-21 | n.a |  |  |
|  |  | IP=52 | IP=26.73(8.89) | IP=35F |  |  |  | GAF |  |  |  |
|  |  | IP+ELA=50 | IP+ELA=25.94(8.34) | IP+ELA=40F |  |  |  |  |  |  |  |
| **Ringel and colleagues (2008) ^45*^** | USA | IBS=10 | ALL (20) =27.6 (10.0) | IBS=10F | An examination of IBS patients and history of abuse in early life of brain functions in response to rectal distension, | History of severe abuse | ROME II criteria and had active symptoms at least over the past 2 weeks. | Physiological distress SCL-90 | n.a |  |  |
|  |  | no-IBS=10 |  | no-IBS=10F |  |  |  |  |  |  |  |
|  |  | Abuse=10 |  | Abuse=10F |  |  |  |  |  |  |  |
|  |  | no-Abuse=10 |  | no-Abuse=10F |  |  |  |  |  |  |  |
|  |  | IBS/abuse=5 |  | IBS/abuse=5F |  |  |  |  |  |  |  |
|  |  | All others=15 |  | All others=15F |  |  |  |  |  |  |  |
| **Skokauskas and colleagues ^30*^** | Ireland | HC=43 | HC=36.16(12) | HC=23F | Assessment of impact of SA on functional imaging markers via an emotional shifting task on MDD patients. | Childhood Trauma Questionnaire (CTQ-SF) | n.a | HRSD | n.a |  |  |
|  |  | MDD+SA=37 | MDD+SA=42.27(11) | MDD+SA=25F |  |  |  | BDI-II |  |  |  |
|  |  | MDD-SA=13 | MDD-SA=38.61(8) | MDD-SA=6F |  |  |  |  |  |  |  |
| **Tozzi and colleagues (2016) ^32^** | Ireland | HC+TA=22 | HC+TA=36.00 (12.32) | HC+TA=22F | An investigation on brain polymorphism association with altered brain structure and function in a cohort of MDD and HC. | CTQ-SF | n.a | HRSD | n.a |  |  |
|  |  | HC+CH=21 | HC+CH=36.43 (14.65) | HC+CH=21F |  |  |  |  |  |  |  |
|  |  | MDD+TA=20 | MDD+TA=45.35 (10.74) | MDD+TA=20F |  |  |  | BDI-II |  |  |  |
|  |  | MDD+CH=20 | MDD+CH=37.80 (9.91) | MDD+CH=20F |  |  |  |  |  |  |  |
| **Tozzi and colleagues (2018) ^33^** | Ireland | CAMI:HC=29 | CAMI:HC=38.28 (12.40) | CAMI:HC=17F | Exploring the association between exposure to childhood in MDD patients and HC with structural and functional differences. | CTQ | n.a | HAM-D | n.a |  |  |
|  |  | CAMI:MDD=31 | CAMI:MDD=40.42 (9.72) | CAMI:MDD=21F |  |  |  |  |  |  |  |
|  |  |  | TCIN:HC=34.00 (11.63) |  |  |  |  |  |  |  |  |
|  |  | TCIN:HC=21 | TCIN:MDD=37.76 (13.17) | TCIN:HC=13F |  |  |  | BDI |  |  |  |
|  |  | TCIN:MDD=25 |  | TCIN:MDD=15F |  |  |  |  |  |  |  |

Suppl. Table 3 Summary Table, demographics for the functional task-based MRI studies*, studies included in the meta-analysis; MDD, Major Depressive Disorder; CP, chronic pain; HC, Healthy Control; ELA, Early Life Adversity; CM, Childhood Maltreatment; CTE, Childhood Trauma Exposure; EAL, Early Adverse Life; ELT, Early Life Trauma; CA, Childhood Abuse; CEM, Childhood Emotional Maltreatment; SA, Sexual Abuse; EA, Emotional Abuse; CECA, Childhood Experience of Care and Abuse; CTQ, Childhood Trauma Questionnaire; CTQ-SF, Childhood Trauma Questionnaire-Short Form; ACEs, Adverse Childhood Experiences; BDI‐II, Beck’s Depression Inventory; QIDS , Quick inventory for depression symptomatology; HAMD, Hamilton depression scale; HAMD-D, Hamilton Rating Scale for Depression; HDRS, Hamilton Depression Rating; MADRS, Montgomery Åsberg Depression Rating Scale (MADRS); IDS-SR, Inventory of Depressive Symptomatology-Self report; HAD, Hospital Anxiety and Depression Scale; SDS, Self-rating Depression Scale; PHQ, Patient Health Questionnaire ; NESDA, Netherlands Study of Depression and Anxiety; GMV, Grey matter volume; IBS, Irritable bowel syndrome; CH, C-homozygous; TA T-allele; ROD, Recent onset Depression; CD, Chronic Depression; ROP, Recent onset Psychosis; CHP, Clinical High-risk to Psychosis; IP, Internalizing Psychopathology; SPD, Somatoform pain disorder; ToM, Theory of Mind; MSPD, Multisomatoform pain disorder; Eriksen flanker task, an fMRI task that allows for testing of both voluntary and nonvoluntary attention

| Author (year) | Structural MRI | |
| --- | --- | --- |
|  | **Contrast of interest** | **Main results** |
| *Studies examining the relationship of MDD or CP compared to Healthy controls* | | |
| *Ahn and colleagues* ^8^ | MDD>HC | Compare with HC, MDD showed increase GMD in the bilateral postcentral gyrus, parieto-occipital cortex, putamen, hippocampus and right thalamus and left cerebellum (declive) and right cerebellum (tuber of vermis). Decrease GMD showed in the bilateral orbitofrontal cortex, middle occipital gyrus and the right dorsomedial prefrontal cortex, right dorsal anterior cingulate cortex, and the left cuneus. |
| *Carballedo and colleagues*^1^ | MDD>HC | Compared with HC, MDD had smaller hippocampal volumes in both hemispheres. Met allele carriers with experiences of childhood adversity, both MDD and HC revealed decreased hippocampal volume. |
| *Frodl and colleagues (2010) Neuropsychopharmacology*^2^ | MDD>HC | Compared to HC, MDD patients with ELA showed decreased hippocampal volumes. |
| *Frodl and colleagues (2010). J Psychiatr Res* ^3^ | MDD>HC | Compared to HC, MDD patients had smaller hippocampal white matter and prefrontal grey matter. MDD with emotional neglect had smaller left hippocampal white matter, for males it was observed in both hemispheres. Physical neglect affected prefrontal grey matter volume in HC. |
| *Frodl and colleagues (2014)*^12^ | MDD>HC | Compared to HC, MDD patients had smaller CA2/3 and CA4/DG. Interactive effect of BDNF allele and CA on CA2/3 and CA4/DG |
| *Frodl and colleagues (2017)* ^13^ | MDD>HC | Independent of MDD, females who had increased exposure to CA were associated with smaller caudate volumes. |
| *Jaworska and colleagues*^19^ | MDD>HC | MDD patients had thicker frontal lobe. Cortical thickness variation in hemispheres on the caudal middle frontal, entorhinal cortex, fusiform cortex, inferior parietal cortex, inferior temporal cortex, isthmus cingulate cortex, lingual cortex, pars orbitalis cortex, pericalcarine cortex, precentral cortex, precuneus cortex, rostral anterior cingulate cortex, rostral middle frontal cortex. |
| *Lenze and colleagues* ^5^ | MDD>HC | MDD patients had decreased volume of the hippocampus. Association between CA with earlier age of depression and with history of recurrent depression. |
| *Lu, S and colleagues*^20^ | MDD>HC | Main effect of diagnosis displays a decrease in the left superior parietal lobe and an increase on the right middle occipital gyrus. Main effect of childhood trauma displays a decrease of the dorsolateral prefrontal cortex. |
| *Lu, X. and colleagues*^21^ | MDD>HC | In the left parahippocampal gyrus, left entorhinal cortex, and left cuneus significant MDD‐by‐CM interactive effects had been found. Compared to HC, MDD patients showed decreased volume on the right middle temporal gyrus. |
| *Opel and colleagues* ^6^ | MDD>HC | Compared to HC, MDD patients showed smaller bilateral hippocampal volumes. |
| *Peng, and colleagues* ^26^ | HC>MDD-CN | Where MDD patients without CN showed decreased WM densities in bilateral sub-lobar extra-nuclear. |
| *Saleh and colleagues* ^7^ | MDD>HC | More ELS exposure was associated with smaller orbito-frontal cortex in MDD patients. MDD patients showed smaller hippocampal volume. Individuals reporting predictive ELSs exhibited smaller volumes of the lateral orbitofrontal cortex and caudate, and decreased cortical thickness in insula bilaterally, left superior frontal, left rostral middle frontal, left inferior parietal-I and -ii, bilateral postcentral, right fusiform and the pericalcarine. |
| *Tozzi and colleagues (2020)* ^34^ | MDD>HC | Banks of the superior temporal sulcus and supramarginal gyrus showed reduction of the cortical thickness and reduction of the middle temporal lobe with association with CM severity. Compared to participants not exposed to CM, participants exposed to CN and CA had cortical thickness reduction in the middle temporal lobe, inferior parietal lobe and precuneus. In males’ participants higher cortical thickness of the rostral anterior cingulate cortex was associated with CM severity, regardless of the diagnosis. |
| *Yang and colleagues* ^41^ | MDD>HC | Compared with HC, MDD patients showed smaller right medial orbitofrontal cortex, left medial prefrontal cortex and left superior parietal lobule. |
| *Yang and colleagues* ^41^ | MDD-CM>HC-CM | The main effect of MDD had been observed for MDD without CM compared to HC without CM, smaller left medial frontal gyrus and right medial rectal gyrus and larger right middle temporal gyrus. |
| *Yuan and colleagues*^43^ | MDD>HC | Compared to HC, MDD patients had smaller amygdala volume. Smaller left CA1 volume in abused MDD compared with no abused MDD had been observed. |
| *Gupta and colleagues (2016)* ^47^ | IBS>HC | Compared to HC, IBS patients demonstrate decreased cortical volume in the bilateral subgenual cingulate cortex. |
| *Meyer and colleagues (2020)* ^46^ | SPD >HC | Compared to HC, SPD patients showed decreased cortical thickness in the right anterior insular. |

Suppl. Table 4 Summary Table of result for the structural MRI studies examining the relationship of MDD or CP compared to Healthy controls. MDD, Major Depressive Disorder; CP, chronic pain; HC, Healthy Control; ELA, Early Life Adversity; CM, Childhood Maltreatment; SA, Sexual Abuse; EA, Emotional Abuse; LS, Life Stress; CECA, Childhood Experience of Care and Abuse; CN, Childhood Neglect; CTQ, Childhood Trauma Questionnaire; CTQ-SF, Childhood Trauma Questionnaire-Short Form; ACEs , Adverse Childhood Experiences; BDI‐II, Beck’s Depression Inventory; QIDS , Quick inventory for depression symptomatology; HAMD, Hamilton depression scale; HAMD-D, Hamilton Rating Scale for Depression; HDRS, Hamilton Depression Rating; MADRS, Montgomery Åsberg Depression Rating Scale (MADRS); IDS-SR, Inventory of Depressive Symptomatology-Self report; HAD, Hospital Anxiety and Depression Scale; SDS, Self-rating Depression Scale; PHQ, Patient Health Questionnaire ; GMV, Grey matter volume; ROD, Recent onset of Depression; IBS, Irritable bowel syndrome; SPD, Somatoform pain disorder

| **Author (year)** | **Selection** | | | | | | | **Comparability** | | **Exposure** | | **Statistical analysis** | | Method of correction |
| --- | --- | --- | --- | --- | --- | --- | --- | --- | --- | --- | --- | --- | --- | --- |
|  | A | | | B | C | D | E | F | G | H | I | J | K |  |
|  | A1 | A2 | A3 |  |  |  |  |  |  |  |  |  |  |  |
| **Ahn and colleagues**^8^ | **+** | **n.a** | **+** | **+** | **+** | **+** | **+** | **+** | **+** | **+** | **-** | **+** | **+** | Monte Carlo |
| **Carballedo and colleagues**^1^ | **+** | **n.a** | **+** | **+** | **+** | **+** | **+** | **+** | **+** | **+** | **-** | **-** | **-** |  |
| **Chaney and colleagues**^9^ | **+** | **n.a** | **+** | **+** | **+** | **+** | **+** | **+** | **+** | **+** | **-** | **+** | **+** | I |
| **Cisler and colleagues**^10^ | **+** | **n.a** | **+** | **+** | **+** | **+** | **-** | **-** | **-** | **+** | **-** | **-** | **-** |  |
| **Colle and colleagues**^11^ | **+** | **n.a** | **?** | **+** | **-** | **-** | **+** | **+** | **+** | **+** | **-** | **-** | **-** |  |
| **Frodl and colleagues (2010) Neuropsychopharmacology**^2^ | **+** | **n.a** | **+** | **+** | **+** | **+** | **+** | **+** | **+** | **+** | **-** | **-** | **+** | I |
| **Frodl and colleagues (2010) J Psychiatr Res**^3^ | **+** | **n.a** | **+** | **+** | **+** | **+** | **+** | **+** | **+** | **+** | **-** | **-** | **-** |  |
| **Frodl and colleagues (2014)** ^12^ | **+** | **n.a** | **+** | **+** | **+** | **+** | **+** | **-** | **+** | **+** | **-** | **-** | **-** |  |
| **Frodl and colleagues (2017)** ^13^ | **+** | **n.a** | **+** | **+** | **+** | **+** | **+** | **-** | **-** | **+** | **-** | **-** | **-** |  |
| **Frost and colleagues**^14^ | **+** | **n.a** | **+** | **+** | **-** | **-** | **-** | **-** | **+** | **+** | **+** | **-** | **?** |  |
| **Gerritsen and colleagues**^4^ | **-** | **n.a** | **+** | **+** | **+** | **?** | **+** | **-** | **-** | **+** | **+** | **-** | **-** |  |
| **Grant and colleagues (2011)** ^15^ | **+** | **n.a** | **+** | **+** | **+** | **+** | **-** | **-** | **+** | **+** | **-** | **-** | **+** | FDR |
| **Grant and colleagues (2014)** ^16^ | **+** | **n.a** | **+** | **+** | **+** | **+** | **-** | **-** | **+** | **+** | **-** | **-** | **+** | FDR |
| **Graziano and colleagues**^17^ | **+** | **n.a** | **+** | **+** | **-** | **-** | **-** | **?** | **?** | **+** | **?** | **-** | **+** | FDR |
| **Gupta and colleagues (2013)** ^48^ | **n.a** | **+** | **+** | **?** | **?** | **?** | **+** | **?** | **?** | **?** | **?** | **?** | **?** |  |
| **Gupta and colleagues (2016)** ^47^ | **n.a** | **+** | **+** | **+** | **+** | **+** | **+** | **+** | **+** | **+** | **?** | **-** | **+** |  |
| **Gupta and colleagues (2019)** ^49^ | **n.a** | **+** | **+** | **+** | **+** | **+** | **+** | **-** | **?** | **+** | **-** | **-** | **+** | FDR |
| **Hentze and colleagues**^18^ | **+** | **n.a** | **+** | **+** | **-** | **-** | **+** | **-** | **?** | **+** | **-** | **-** | **+** | I |
| **Jaworska and colleagues**^19^ | **+** | **n.a** | **+** | **+** | **+** | **+** | **+** | **-** | **-** | **+** | **+** | **-** | **-** |  |
| **Lenze and colleagues**^5^ | **+** | **n.a** | **+** | **+** | **+** | **+** | **+** | **-** | **?** | **+** | **-** | **-** | **-** |  |
| **Lu, S and colleagues**^20^ | **+** | **n.a** | **+** | **+** | **+** | **+** | **+** | **+** | **+** | **+** | **-** | **+** | **?** |  |
| **Lu, X. and colleagues**^21^ | **+** | **n.a** | **+** | **+** | **+** | **+** | **+** | **+** | **+** | **+** | **+** | **+** | **-** |  |
| **Meyer and colleagues**^46^ | **n.a** | **+** | **+** | **+** | **+** | **+** | **-** | **+** | **+** | **+** | **+** | **-** | **-** |  |
| **Miller and colleagues**^22^ | **+** | **n.a** | **+** | **-** | **-** | **-** | **+** | **-** | **+** | **+** | **-** | **+** | **+** | I |
| **Monninger and colleagues**^23^ | **+** | **n.a** | **+** | **+** | **+** | **+** | **+** | **-** | **-** | **+** | **+** | **-** | **-** |  |
| **Noll-Hussong and colleagues**^44^ | **?** | **+** | **+** | **?** | **+** | **+** | **-** | **+** | **+** | **+** | **-** | **-** | **-** |  |
| **Ohashi and colleagues**^24^ | **-** | **n.a** | **+** | **+** | **+** | **+** | **+** | **-** | **?** | **+** | **?** | **-** | **?** |  |
| **Opel and colleagues**^6^ | **+** | **n.a** | **+** | **+** | **+** | **+** | **+** | **+** | **+** | **+** | **-** | **+** | **+** |  |
| **Oshri and colleagues**^25^ | **-** | **n.a** | **+** | **+** | **?** | **-** | **+** | **?** | **?** | **+** | **+** | **-** | **-** |  |
| **Peng, and colleagues**^26^ | **+** | **n.a** | **+** | **+** | **+** | **+** | **+** | **+** | **-** | **+** | **?** | **-** | **-** |  |
| **Peters and colleagues**^27^ | **-** | **n.a** | **+** | **+** | **+** | **?** | **+** | **+** | **+** | **+** | **-** | **+** | **+** | I |
| **Ringel and colleagues**^45^ | **n.a** | **+** | **+** | **?** | **+** | **+** | **-** | **?** | **-** | **+** | **+** | **-** | **+** |  |
| **Saleh and colleagues** ^7^ | **+** | **n.a** | **+** | **+** | **+** | **+** | **+** | **-** | **+** | **+** | **+** | **-** | **-** |  |
| **Salokangas and colleagues**^30^ | **?** | **n.a** | **+** | **+** | **-** | **+** | **+** | **+** | **+** | **+** | **-** | **-** | **-** |  |
| **Sara and colleagues** ^29^ | **+** | **n.a** | **+** | **+** | **+** | **+** | **+** | **-** | **+** | **+** | **-** | **-** | **-** |  |
| **Skokauskas and colleagues** ^28^ | **+** | **n.a** | **+** | **?** | **+** | **+** | **-** | **+** | **?** | **+** | **-** | **-** | **+** | FDR |
| **Tatham and colleagues** ^31^ | **+** | **n.a** | **+** | **+** | **+** | **+** | **+** | **?** | **+** | **+** | **-** | **-** | **+** | I |
| **Tozzi and colleagues (2016)** ^32^ | **+** | **n.a** | **+** | **+** | **+** | **?** | **+** | **-** | **?** | **+** | **?** | **-** | **+** | FEW |
| **Tozzi and colleagues (2018)** ^33^ | **+** | **n.a** | **+** | **+** | **+** | **+** | **+** | **+** | **?** | **+** | **+** | **-** | **+** | FDR |
| **Tozzi and colleagues (2020)** ^34^ | **+** | **n.a** | **+** | **+** | **+** | **+** | **+** | **-** | **?** | **+** | **-** | **+** | **+** |  |
| **Ugwu** **and colleagues** ^8^ | **+** | **n.a** | **+** | **+** | **+** | **+** | **+** | **+** | **+** | **+** | **?** | **-** | **?** |  |
| **van Harmelen and colleagues** ^36^ | **+** | **n.a** | **+** | **+** | **+** | **+** | **+** | **+** | **+** | **+** | **+** | **+** | **+** |  |
| **Vythilingam and colleagues** ^37^ | **+** | **n.a** | **+** | **+** | **+** | **?** | **-** | **-** | **+** | **+** | **-** | **+** | **-** |  |
| **Wang and colleagues**^38^ | **+** | **n.a** | **+** | **+** | **+** | **+** | **+** | **?** | **?** | **+** | **?** | **-** | **+** | Monte Carlo |
| **Wu and colleagues** ^39^ | **+** | **n.a** | **+** | **+** | **+** | **+** | **+** | **-** | **-** | **+** | **-** | **-** | **+** | FDR |
| **Xu and colleagues** ^40^ | **+** | **n.a** | **+** | **+** | **+** | **+** | **-** | **+** | **+** | **+** | **+** | **+** | **-** |  |
| **Yang and colleagues** ^41^ | **+** | **n.a** | **+** | **+** | **+** | **+** | **+** | **-** | **+** | **+** | **-** | **+** | **+** |  |
| **Yu and colleagues** ^42^ | **+** | **n.a** | **+** | **+** | **+** | **+** | **+** | **+** | **+** | **+** | **+** | **-** | **-** |  |
| **Yuan and colleagues** ^43^ | **+** | **n.a** | **?** | **+** | **+** | **+** | **+** | **+** | **+** | **+** | **+** | **-** | **-** |  |

Suppl. Table 5 Risk of Bias Assessment. A represents the adequate definition of A1-MDD, A2-CP and A3-ELA; B, Representativeness; C, Controls; D, Definition of controls; E, Study group; F, Age & Gender; G, Other variables; H, Same exposure; I, Drop-out rate; J, p value < 0.001; K, corrections; Method of correction, Method used for correcting of multiple testing at each voxel.

| **MDD or CP or HC: with ACEs (n = 139) > without ACEs (n=217)** ^22,27,30 ,44,45^ | | |
| --- | --- | --- |
| **Study-Group** | **Contrast** | **Number of Participants** |
| **Miller and colleagues**^22^ | 2x(MDD+CM>MDD-CM) |  |
| MDD+CM |  | 43 |
| MDD-CM |  | 55 |
| **Peter and colleagues**^27^ | 2x(IP+ELA>IP+HC) |  |
| IP+ELA |  | 50 |
| IP+HC |  | 82 |
| **Skokauskas and colleagues**^30^ | 2x(MDD+SA>MDD-SA) |  |
| MDD+SA |  | 13 |
| MDD-SA |  | 37 |
| **Grant and colleagues**^15^ | MDD+CT>MDD |  |
| MDD+CT |  | 10 |
| MDD |  | 10 |
| **Noll-Hussong and colleagues**^44^ | Abuse>No Abuse |  |
| Abuse |  | 8 |
| No Abuse |  | 8 |
| **Ringel and colleagues**^,45^ | Abuse>No Abuse |  |
| Abuse |  | 10 |
| No Abuse |  | 10 |
|  | IBS+Abuse>Others |  |
| IBS+Abuse |  | 5 |
| Others |  | 15 |

Suppl. Table 6 fMRI meta-analysis group of MDD or CP or HC: with ACEs > without ACEs participants. Major Depressive Disorder; CP, chronic pain; HC, Healthy Control; ELA, Early Life Adversity; CM, Childhood Maltreatment; SA, Sexual Abuse; CT, Childhood Trauma; ACEs, Adverse Childhood Experiences; IBS, Irritable bowel syndrome; IP, Internalizing Psychopathology

# References

1. Carballedo A, Morris D, Zill P, et al. Brain-derived neurotrophic factor val66met polymorphism and early life adversity affect hippocampal volume. *American Journal of Medical Genetics Part B-Neuropsychiatric Genetics*. 2013; 162(2):183–190.
2. Frodl T, Reinhold E, Koutsouleris N, et al. Childhood stress, serotonin transporter gene and brain structures in major depression. *Neuropsychopharmacology*. 2010; 35(6):1383–1390.
3. Frodl T, Reinhold E, Koutsouleris N, et al. Interaction of childhood stress with hippocampus and prefrontal cortex volume reduction in major depression. *J Psychiatr Res*. 2010; 44(13):799–807.
4. Gerritsen L, van Velzen L, Schmaal L, et al. Childhood maltreatment modifies the relationship of depression with hippocampal volume. *Psychol Med*. 2015; 45(16):3517–26.
5. Lenze S. N, Xiong C, and Sheline Y. I. Childhood adversity predicts earlier onset of major depression but not reduced hippocampal volume. *Psychiatry ResearchNeuroimaging*. 2008; 162(1):39–49.
6. Opel N, Redlich R, Zwanzger P, et al. Hippocampal atrophy in major depression: a function of childhood maltreatment rather than diagnosis? *Neuropsychopharmacology*. 2014; 39(12):2723–2731.
7. Saleh A, Potter G. G, McQuoid D. R, et al. Effects of early life stress on depression, cognitive performance and brain morphology. *Psychol Med*. 2017; 47(1):171–181.
8. Ahn S. J, Kyeong S, Suh S. H, et al. What is the impact of child abuse on gray matter abnormalities in individuals with major depressive disorder: a case control study. *Bmc Psychiatry*. 2016; 16.
9. Chaney A, Carballedo A, Amico F, et al. Effect of childhood maltreatment on brain structure in adult patients with major depressive disorder and healthy participants. *Journal of Psychiatry & Neuroscience*. 2014; 39(1):50–59.
10. Cisler J. M, James G. A, Tripathi S, et al. Differential functional connectivity within an emotion regulation neural network among individuals resilient and susceptible to the depressogenic effects of early life stress. *Psychol Med*. 2013; 43(3):507–18.
11. Colle R, Segawa T, Chupin M, et al. Early life adversity is associated with a smaller hippocampus in male but not female depressed in-patients: a case-control study. *Bmc Psychiatry*. 2017; 17.
12. Frodl T, Skokauskas N, Frey E. M, et al. Bdnf val66met genotype interacts with childhood adversity and influences the formation of hippocampal subfields. *Human Brain Mapping*. 2014; 35(12):5776–5783.
13. Frodl T, Janowitz D, Schmaal L, et al. Childhood adversity impacts on brain subcortical structures relevant to depression. *Journal of Psychiatric Research*. 2017; 86:58–65.
14. Frost C. P, Meyerand M. E, Birn R. M, et al. Childhood emotional abuse moderates associations among corticomotor white matter structure and stress neuromodulators in women with and without depression. *Frontiers in Neuroscience*. 2018;

12.

1. Grant M. M, Cannistraci C, Hollon S. D, et al. Childhood trauma history differentiates amygdala response to sad faces within mdd. *J Psychiatr Res*. 2011; 45(7):886–95.
2. Grant M. M, White D, Hadley J, et al. Early life trauma and directional brain connectivity within major depression. *Hum Brain Mapp*. 2014; 35(9):4815–26.
3. Graziano R. C, Bruce S. E, Paul R. H, et al. The effects of bullying in depression on white matter integrity. *Behav Brain Res*. 2019; 363:149–154.
4. Hentze C, Walter H, Schramm E, et al. Functional correlates of childhood maltreatment and symptom severity during affective theory of mind tasks in chronic depression. *Psychiatry Res Neuroimaging*. 2016; 250:1–11.
5. Jaworska N, MacMaster F. P, Gaxiola I, et al. A preliminary study of the influence of age of onset and childhood trauma on cortical thickness in major depressive disorder. *Biomed Research International*. 2014; 2014.
6. Lu S, Xu R, Cao J, et al. The left dorsolateral prefrontal cortex volume is reduced in adults reporting childhood trauma independent of depression diagnosis.

*J Psychiatr Res*. 2019; 112:12–17.

1. Lu X. W, Guo H, Sun J. R, et al. A shared effect of paroxetine treatment on gray matter volume in depressive patients with and without childhood maltreatment: A voxel-based morphometry study. *CNS Neurosci Ther*. 2018; 24(11):1073–1083.
2. Miller S, McTeague L. M, Gyurak A, et al. Cognition-childhood maltreatment interactions in the prediction of antidepressant outcomes in major depressive disorder patients: Results from the ispot-d trial. *Depress Anxiety*. 2015; 32(8):594–604.
3. Monninger M, Kraaijenvanger E. J, Pollok T. M, et al. The long-term impact of early life stress on orbitofrontal cortical thickness. *Cerebral Cortex*. 2020; 30(3):1307–1317.
4. Ohashi K, Anderson C. M, Bolger E. A, et al. Childhood maltreatment is associated with alteration in global network fiber-tract architecture independent of history of depression and anxiety. *Neuroimage*. 2017; 150:50–59.
5. Oshri A, Gray J. C, Owens M. M, et al. Adverse childhood experiences and amygdalar reduction: High-resolution segmentation reveals associations with subnuclei and psychiatric outcomes. *Child Maltreatment*. 2019; 24(4):400–410.
6. Peng H, Ning Y, Zhang Y, et al. White-matter density abnormalities in depressive patients with and without childhood neglect: a voxel-based morphometry (vbm) analysis. *Neurosci Lett*. 2013; 550:23–8.
7. Peters A. T, Burkhouse K. L, Kinney K. L, and Phan K. L. The roles of early-life adversity and rumination in neural response to emotional faces amongst anxious and depressed adults. *Psychological Medicine*. 2019; 49(13):2267–2278.
8. Salokangas R. K. R, Hietala J, Armio R. L, et al. Effect of childhood physical abuse on social anxiety is mediated via reduced frontal lobe and amygdalahippocampus complex volume in adult clinical high-risk subjects. *Schizophr Res*. 2020; 24(20):30295–4.
9. Sara P, Veronica A, Silvia B, et al. Impact of early and recent stress on white matter microstructure in major depressive disorder. *Journal of Affective Disorders*. 2018; 225:289–297.
10. Skokauskas N, Carballedo A, Fagan A, and Frodl T. The role of sexual abuse on functional neuroimaging markers associated with major depressive disorder. *World Journal of Biological Psychiatry*. 2015; 16(7):513–520.
11. Tatham E. L, Ramasubbu R, Gaxiola-Valdez I, et al. White matter integrity in major depressive disorder: Implications of childhood trauma, 5-httlpr and bdnf polymorphisms. *Psychiatry Res Neuroimaging*. 2016; 253:15–25.
12. Tozzi L, Carballedo A, Wetterling F, et al. Single-nucleotide polymorphism of the fkbp5 gene and childhood maltreatment as predictors of structural changes in brain areas involved in emotional processing in depression. *Neuropsychopharmacology*. 2016; 41(2):487–497.
13. Tozzi L, Farrell C, Booij L, et al. Epigenetic changes of fkbp5 as a link connecting genetic and environmental risk factors with structural and functional brain changes in major depression. *Neuropsychopharmacology*. 2018; 43(5):1138–1145.
14. Tozzi L, Garczarek L, Janowitz D, et al. Interactive impact of childhood maltreatment, depression, and age on cortical brain structure: mega-analytic findings from a large multi-site cohort. *Psychol Med*. 2020; 50(6):1020–1031.
15. Ugwu I. D, Amico F, Carballedo A, et al. Childhood adversity, depression, age and gender effects on white matter microstructure: a dti study. *Brain Structure & Function*. 2015; 220(4):1997–2009.
16. van Harmelen A. L, van Tol M. J, van der Wee N. J, et al. Reduced medial prefrontal cortex volume in adults reporting childhood emotional maltreatment. *Biol Psychiatry*. 2010; 68(9):832–8.
17. Vythilingam M, Heim C, Newport J, et al. Childhood trauma associated with smaller hippocampal volume in women with major depression. *Am J Psychiatry*. 2002; 159(12):2072–80.
18. Wang L. F, Dai Z. J, Peng H. J, et al. Overlapping and segregated resting-state functional connectivity in patients with major depressive disorder with and without childhood neglect. *Human Brain Mapping*. 2014; 35(4):1154–1166.
19. Wu H, Wu C, Wu F, et al. *Covariation between Childhood-Trauma Related RestingState Functional Connectivity and Affective Temperaments is Impaired in Individuals with Major Depressive Disorder*. Neuroscience. 453 (pp 102-112), 2021. Date of Publication: 15 Jan 2021.; 2021.
20. Xu Z. X, Zhang J, Wang D, et al. Altered brain function in drug-naive major depressive disorder patients with early-life maltreatment: A resting-state fmri study.

*Frontiers in Psychiatry*. 2019; 10.

1. Yang S, Cheng Y, Mo Y, et al. Childhood maltreatment is associated with gray matter volume abnormalities in patients with first-episode depression. *Psychiatry Res Neuroimaging*. 2017; 268:27–34.
2. Yu M, Linn K. A, Shinohara R. T, et al. Childhood trauma history is linked to abnormal brain connectivity in major depression. *Proceedings of the National Academy of Sciences*. 2019; 116(17):8582–8590.
3. Yuan M. L, Rubin-Falcone H, Lin X. J, et al. Smaller left hippocampal subfield ca1 volume is associated with reported childhood physical and/or sexual abuse in major depression: A pilot study. *Journal of Affective Disorders*. 2020; 272:348–354.
4. Noll-Hussong M, Otti A, Laeer L, et al. Aftermath of sexual abuse history on adult patients suffering from chronic functional pain syndromes: An fmri pilot study. *Journal of Psychosomatic Research*. 2010; 68(5):483–487.
5. Ringel Y, Drossman D. A, Leserman J. L, et al. Effect of abuse history on pain reports and brain responses to aversive visceral stimulation: An fmri study. *Gastroenterology*. 2008; 134(2):396–404.
6. Meyer E, Morawa E, Nacak Y, et al. Insular cortical thickness in patients with somatoform pain disorder: Are there associations with symptom severity and childhood trauma? *Frontiers in psychiatry*. 2020; 11:497100–497100.
7. Gupta A, Labus J, Kilpatrick L. A, et al. Interactions of early adversity with stressrelated gene polymorphisms impact regional brain structure in females. *Brain Struct Funct*. 2016; 221(3):1667–79.
8. Gupta A, Kilpatrick L. A, Braun A, et al. *Early adverse life events: Influence on resting state connectivity in somatosensory, cognitive and pain regions in male and female patients with irritable bowel syndrome*. Gastroenterology. Conference: Digestive Disease Week 2013, DDW 2013. Orlando, FL United States. Conference Publication: (var.pagings). 144 (5 SUPPL. 1) (pp S150-S151), 2013. Date of Publication: May 2013.; 2013.
9. Gupta A, Bhatt R. R, Naliboff B. D, et al. Impact of early adverse life events and sex on functional brain networks in patients with urological chronic pelvic pain syndrome (ucpps): A mapp research network study. *Plos One*. 2019; 14(6).
